# Crystal Composition Transformer: Self‐Learning Neural Language Model for Generative and Tinkering Design of Materials

**DOI:** 10.1002/advs.202304305

**Published:** 2024-08-05

**Authors:** Lai Wei, Qinyang Li, Yuqi Song, Stanislav Stefanov, Rongzhi Dong, Nihang Fu, Edirisuriya M. D. Siriwardane, Fanglin Chen, Jianjun Hu

**Affiliations:** ^1^ Department of Computer Science and Engineering University of South Carolina Columbia SC 29201 USA; ^2^ Department of Computer Science University of Southern Maine Portland ME 04131 USA; ^3^ Department of Physics University of Colombo Colombo 00300 Sri Lanka; ^4^ Department of Mechanical Engineering University of South Carolina Columbia SC 29201 USA

**Keywords:** blank filling, deep learning, language models, materials discovery, materials generator

## Abstract

Self‐supervised neural language models have recently achieved unprecedented success from natural language processing to learning the languages of biological sequences and organic molecules. These models have demonstrated superior performance in the generation, structure classification, and functional predictions for proteins and molecules with learned representations. However, most of the masking‐based pre‐trained language models are not designed for generative design, and their black‐box nature makes it difficult to interpret their design logic. Here a Blank‐filling Language Model for Materials (BLMM) Crystal Transformer is proposed, a neural network‐based probabilistic generative model for generative and tinkering design of inorganic materials. The model is built on the blank‐filling language model for text generation and has demonstrated unique advantages in learning the “materials grammars” together with high‐quality generation, interpretability, and data efficiency. It can generate chemically valid materials compositions with as high as 89.7% charge neutrality and 84.8% balanced electronegativity, which are more than four and eight times higher compared to a pseudo‐random sampling baseline. The probabilistic generation process of BLMM allows it to recommend materials tinkering operations based on learned materials chemistry, which makes it useful for materials doping. The model is applied to discover a set of new materials as validated using the Density Functional Theory (DFT) calculations. This work thus brings the unsupervised transformer language models based generative artificial intelligence to inorganic materials. A user‐friendly web app for tinkering materials design has been developed and can be accessed freely at www.materialsatlas.org/blmtinker.

## Graphical abstract

A novel Blank‐filling Language Model for Materials (BLMM) Crystal Transformer is introduced for the generative design of inorganic materials. This probabilistic model demonstrates high data efficiency, interpretability, and superior performance in generating chemically valid compositions with balanced charge and electronegativity. BLMM excels in interpreting design logic and recommending tinkering operations, advancing AI‐driven materials discovery.

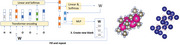

## 1 Introduction

The discovery of novel functional materials such as high‐capacity and safe electrodes and electrolytes for batteries or room‐temperature superconductors has the potential to transform diverse industries.^[^ ^1^, ^2^ ^]^ However, due to the sophisticated relationships of material composition‐structure‐properties, centuries of rational design strategies have only covered an extremely limited chemical design space, among which the screening‐based approaches for discovering new materials are constrained by the limited scale and lack of diversity of known materials. Historically, most known materials were discovered via trial‐and‐error approaches. Many materials are the result of the tinkering process on existing materials, which is however, impeded by the incomplete understanding of the function‐related mechanisms and factors.^[^ ^3^ ^]^ To address these challenges, researchers have been exploring various computational and data‐driven approaches. For example, high‐throughput computational screening methods have been developed to accelerate materials discovery.^[^ ^4^, ^5^ ^]^ These methods have led to the creation of large materials databases such as the Materials Project,^[^ ^6^ ^]^ OQMD,^[^ ^7^ ^]^ and Aflowlib,^[^ ^8^ ^]^ which have become invaluable resources for materials scientists. Fractional element substitution is the standard approach in material doping, which is usually required to fine‐tune the electronic properties of semiconductors^[^ ^9^ ^]^ and other energy materials.^[^ ^10^ ^]^ In addition, element substitutions over existing crystal structures have been routinely used nowadays to generate new hypothetical materials,^[^ ^7^, ^8^, ^11^ ^]^ which are commonly used in generating hypothetical materials in Materials Project, OQMD, and Aflowlib databases.

With the progress of generative machine learning, generative adversarial networks (GAN) and variational autoencoders (VAEs) could be trained to generate chemically valid material compositions.^[^ ^12^, ^13^, ^14^ ^]^ These deep learning‐based composition generators can achieve much higher efficiency in sampling chemically valid compositions compared to random algorithm or exhaustive substitution methods. Graph neural networks have shown promise in predicting material properties and generating new structures.^[^ ^15^, ^16^ ^]^ These approaches complement the GAN‐based and VAEs‐based methods and offer different perspectives on the materials generation problem. While these composition generators themselves do not generate the structures, they can be combined with modern crystal structure prediction (CSP) algorithms^[^ ^17^ ^]^ for structure determination. This composition generator combined with CSP is especially promising due to the emergence of modern template‐based CSP algorithms,^[^ ^18^, ^19^ ^]^ in which it has been shown that a large percentage of crystal structures can be predicted using known crystal prototypes.^[^ ^20^, ^21^ ^]^ However, the black‐box nature of the deep neural network‐based generator makes it difficult to interpret the black‐box GAN and VAEs models in terms of the chemical knowledge they learn and how they exploit the learned implicit chemical knowledge for composition generation. On the other hand, materials tinkering or doping is one of the most widely used approaches to explore new materials.^[^ ^3^, ^22^, ^23^ ^]^ During these processes, material scientists usually resort to their intuition, chemical knowledge, and expertise to select substitution or doping elements and proportions to tune the properties of the material^[^ ^10^, ^24^, ^25^, ^26^, ^27^ ^]^ by considering a variety of factors such as compatibility of oxidation states, charge neutrality, coordination number, atomic radius, and other heuristic knowledge. Recent advancements in explainable AI (XAI) techniques^[^ ^28^, ^29^ ^]^ offer potential solutions to the interpretability challenge of deep learning models in materials science. These techniques could be applied to understand the decision‐making process of generative models, providing insights into the learned chemical rules and relationships. Margraf et al.^[^ ^30^ ^]^ suggested that “materials grammars” can be defined based on expert knowledge to narrow down the design space in generative materials design. However, it is challenging for humans to explicitly enumerate all such chemical grammar rules considering so many chemical context‐based dependencies among elements of stable compounds. To address this issue, models with the capability to automatically distill chemical knowledge from data are highly desirable as shown in both language‐learning intelligent machines^[^ ^31^ ^]^ and AI game‐players such as AlphaZero.^[^ ^32^ ^]^

With the rapid growth of automatically pre‐trained self‐supervised learning models, such as Bidirectional Encoder Representations from Transformers (BERT)^[^ ^33^ ^]^ and Generative Pretrained Transformer (GPT) Inorganic Crystal Structure Database,^[^ ^34^ ^]^ have proven effective at learning language grammars^[^ ^31^ ^]^ for text generation,^[^ ^35^, ^36^ ^]^ achieving superior performance in many downstream tasks, including reading comprehension and question–answering. These language models have been further adapted to domains such as proteins,^[^ ^37^, ^38^ ^]^ and organic molecules.^[^ ^39^, ^40^, ^41^, ^42^, ^43^, ^44^, ^45^ ^]^ Alley et al.^[^ ^46^ ^]^ showed that self‐supervised protein language models are effective at learning protein representations for downstream tasks such as solubility prediction. Building on this, Brandes et al. proposed ProteinBert^[^ ^37^ ^]^ which showed strong performance in a variety of protein property prediction tasks, including protein structure and post‐translational modifications prediction, using the learned protein representation. Kim et al.^[^ ^42^ ^]^ combined a transformer encoder with a conditional variational autoencoder (cVAE) to achieve high‐performance molecule generation. Dollar et al.^[^ ^47^ ^]^ introduced attention‐based generative models with a β‐VAE for de novo molecular design to learn a molecular strings' relationship. Nevertheless, almost all existing language models for protein or molecule generation thus far function mainly as black boxes, without providing interpretable explanations of the learned grammar or rules. They also face challenges in incorporating domain knowledge, as these generative language models do not explicitly model the generative process. In the field of materials science, natural language processing (NLP) techniques and pre‐trained language models have been successfully applied to extract knowledge from scientific literature for materials discovery. Tshitoyan et al.^[^ ^48^ ^]^ demonstrated that unsupervised word embedding models trained on materials science abstracts can capture complex materials science concepts and even predict new materials years before their discovery. This work highlights the potential of language models in capturing and representing materials knowledge. LM Antunes et al.^[^ ^49^ ^]^ use language models to generate crystal structures as discrete sequences by training from scratch on millions of CIF strings. Gupta et al.^[^ ^50^ ^]^ proposed MatSciBERT for text mining and information extraction, trained on a large corpus of peer‐reviewed materials science publications. Dagdelen et al.^[^ ^51^ ^]^ utilized pre‐trained large language models (GPT‐3, Llama‐2) are fine‐tuned on a few hundred training examples are capable of extracting structured knowledge from unstructured text and formatting the information in user‐defined schemas. However, these methods are not intended for generating new materials and do not model the dependency relationships of the elements within material compositions. Mask prediction‐based language models have been used to learn latent representations of elements, as demonstrated in the Atom2vec method.^[^ ^52^ ^]^ Unsupervised word embedding learning has also shown capability in capturing latent materials knowledge from materials science literature, efficiently encoding this knowledge as information‐dense word embeddings (vector representations of words) without human labeling or supervision.^[^ ^48^ ^]^ These learned embeddings can capture complex materials science concepts such as the underlying structure of the periodic table and structure‐property relationships in materials. While these recent advances demonstrate the potential of language models in materials science, there remains a notable gap in research specifically applying deep language models to the generation of inorganic materials compositions.

Inspired by the transformer‐based language models with their grammar/rule learning capability, state‐of‐the‐art performance on structure and function predictions, and especially the capability for text/protein/molecule sequence generation, here we propose BLMM, a self‐supervised language modeling approach^[^ ^53^ ^]^ for the generative and tinkering design of inorganic materials compositions. Our composition generation models can be used to generate chemically valid formulas, which can then be fed to modern crystal structure prediction algorithms^[^ ^18^, ^19^, ^54^, ^55^, ^56^, ^57^ ^]^ to generate and screen out a large number of hypothetical crystal structures. This integration of composition generation with structure prediction represents a significant advancement in the field of computational materials discovery, potentially accelerating the identification of novel materials with desired properties. Our BLMM crystal transformer is based on a special self‐supervised blank‐filling language model (BLM) for text generation,^[^ ^53^ ^]^ but is trained with materials composition/formula data in the form of unlabeled expanded element symbol sequences sorted by the element electronegativity. These materials composition sequences use a small vocabulary of 118 or fewer elements, which is much larger than the 20 amino acid elements in protein language models but is much smaller than the vocabulary of natural texts. Unlike natural language texts, materials composition sequences have strong constraints among the elements due to the requirements to form chemically valid and structurally stable structures, which involve complex atomic interactions from ionic or covalent bonds and oxidation states of constituent elements. Effective generation models thus are required to learn complex local and long‐range dependencies and contexts that the transformer neural network models excel at detecting and modeling. Our probabilistic blank‐filling model has an advantage over the heuristic or data mining element substitution models^[^ ^58^, ^59^ ^]^ as it can consider the chemical context within the formulas rather than only element property compatibility. This approach aligns with recent trends in materials informatics that emphasize the importance of capturing complex relationships and dependencies in materials data.^[^ ^60^, ^61^ ^]^ Our extensive de novo materials composition generation and materials tinkering show that our BLM‐based materials generators learn chemical grammars and achieve interpretable generation due to their probabilistic predictions of the generation actions/steps. This interpretability aspect addresses a significant challenge in the field of AI‐driven materials discovery, where the lack of explainability has been a concern for researchers and practitioners.^[^ ^62^, ^63^ ^]^ Our contributions can be summarized as follows:

1. We have developed a composition generative model capable of inventing new hypothetical materials formulas by learning chemical rules such as charge neutrality, electronegativity balance, and other hidden patterns.
2. Our generative model can be utilized for the design and tinkering of known materials, effectively replacing or assisting the heuristic methods used by materials experts.
3. Our model has the capability to generate novel materials that extend beyond the known structure prototypes typically used in element‐substitution methods.
4. Our model is designed to integrate seamlessly with state‐of‐the‐art crystal structure prediction software and algorithms, thus facilitating a more comprehensive approach to new materials discovery.
5. We provided open‐source code and a user‐friendly composition tinkering website to promote widespread adoption and further advancement in the field, w. These resources are invaluable tools for the broader research community, encouraging collaboration and accelerating progress in materials science.
6. The significance of composition generative models is underscored by the abundance of research on crystal structure prediction algorithms, all of which require an initial composition as input. Our work addresses this crucial first step in the materials discovery process, potentially accelerating the entire pipeline from composition generation to structure prediction and property evaluation.

## 2 Results

### 2.1 Generative and Tinkering Materials Design as a Blank‐Filling Process

A typical ternary material composition can be represented as *A*_*x*_ *B*_*y*_ *C*_*z*_ where A/B/C are elements and x/y/z are the number of atoms of corresponding elements. The same rule applies to compounds with different number of elements. If we only consider the cases where x/y/z are integers, we can expand the formula into *A*_1_ *A*_2_…*A*_*x*_ *B*_1_ *B*_2_…*B*_*y*_ *C*_1_ *C*_2_..*C*_*z*_. For example, *SrTiO*_3_ can be expanded to *Sr* *Ti* *O* *O* *O*, which becomes a regular sequence similar to a natural text sequence of words, a sequence of amino acids, or a SMILES representation of a molecule.

Generating a chemical formula *SrTiO*_3_ as represented by its expanded element sequence *Sr* *Ti* *O* *O* *O* from scratch can be done by the following canvas rewriting process (**Table** **1**): it starts with a starting canvas with a single blank #1 _ (non‐terminal). For each blank, there are four possible canvas replacement/rewriting actions for each possible element out of 118 elements (or a subset): 1) action E: replace a blank with element E; 2) action _E: replace a blank with element E and insert a new blank on its left side, allowing further element insertion; 3) action E_: replace a blank with element E and insert a new blank on its right side, allowing further element insertion; 4) action _E_: replace blank with element E and insert new blanks on both sides. In Table 1, step 1 selects the action _E_ with element Ti, it generates a canvas with two blanks _Ti_. The model then selects action E, which just replaces the left blank with element Sr. The next two steps all select action E_, which replaces the blank with element O and inserts a blank on the right. The final step just replaces the blank with another oxygen element. In addition to generation from scratch, the above canvas rewriting process can be naturally used for materials tinkering: we only need to mask some of the atoms in a known material formula as blanks, and the blank‐filling process works the same way as de novo generation.

**Table 1:** Composition generation as a canvas rewriting process.

Canvas rewriting with four actions: (E, _E, E_, _E_)		
Step t	Action	Operation
0. #1	_E_	Replace #1 blank with _Ti_
1. #1 Ti #2	E	Replace #1 blank with Sr
2. Sr Ti #1	E_	Replace #1 blank with O_
3. Sr Ti O #1	E_	Replace #1 blank with O_
4. Sr Ti O O #1	E	Replace #1 blank with O
5. Sr Ti O O O		

The key for machine learning to learn the blank‐filling generative model is how to learn the dependency of the rewriting process (blank‐filling) over the preexisting contexts from the corpse of known inorganic materials composition sequences. The conditional developmental process of the canvas rewriting is similar to the growth of the body plan of living organisms based on the cellular sense of growth factor or morphogen gradient^[^ ^64^ ^]^ in a local cellular context. The rewriting process has also been modeled in the synthesis of circuits and dynamic systems^[^ ^65^ ^]^ using genetic programming. Here we use a transformer‐based blank‐filling language model to learn the context‐based material composition generation process.^[^ ^53^ ^]^

### 2.2 Crystal Transformer: Blank Filling Language Model for Materials Composition Generation

Our generative design model is based on the text‐filling blank language model (BLM),^[^ ^53^ ^]^ which is different from other popular deep language models such as BERT^[^ ^33^ ^]^ and XL‐Net,^[^ ^66^ ^]^ which usually mask and predict 15% of tokens conditioned on the remaining text. This strategy is great for representation learning but may not be optimal for sequence generation. The BLM model directly models the probabilistic dependency of words within the sentences and uses it to guide the sentence generation or blank‐filling process, which makes it capable to fill blanks with partially specified text and achieve fine‐grain control of generation locations while respecting the preceding and following contexts. It also has the capability of filling a variable number of missing tokens.

**Figure 1:**
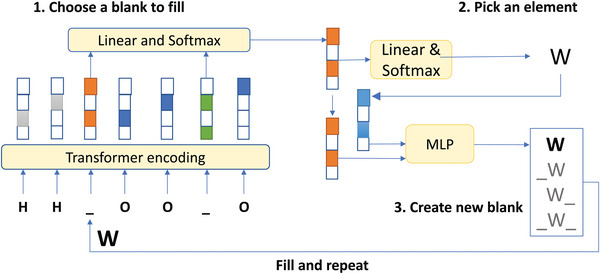
Neural network architecture of the blank‐filling language model BLMM for material tinkering using H_2_WO_4_ as an example.

The architecture of our blank language model for materials (BLMM) is shown in **Figure** **1**. It consists of four main networks including the transformer network which encodes each of the tokens from the input, the expanded material formula with masked blanks, into position and semantic dependent embeddings. Then a blank selection network composed of linear and softmax layers will decide which blank to fill first. Next, the element selection network, also composed of linear and softmax layers, will pick an appropriate element to fill the selected blank. The embedding of the selected blank and the embedding of the picked element are then concatenated together and fed to the multi‐layer Perceptron network to decide one out of four possible blank insertion options. Once the option is made, the canvas will be updated using the newly selected element and inserted blanks and the process will be repeated until all blanks have been filled.

The training process works as follows: first randomly pick a training formula *x* = (*x*_1_, ⋅⋅⋅, *x*_*n*_); then randomly sample *t* between 0 and *n* − 1 and sample an *n*‐permutation σ, which indicates the generation order of the elements in the given formula. Now construct a canvas *c* that keeps the first *t* tokens $x_{\sigma_{j}}$ (*j* = 1,…, *t*) and collapses the remaining *n* − *t* tokens as blanks. Next, get *n* − *t* target actions *a*_*j* − *t*_ for filling $x_{\sigma_{j}}$ (*j* = *t* + 1,…, *n*) into a canvas. Then we compute the training loss by feeding the canvas *c* into the neural networks and get the probabilities to pick the above‐determined actions.

The procedure has a large variance and can only compute the loss of a single action in one pass if the loss is computed by Equation (1), achieved by applying Jensen's inequality to lower bound the log‐likelihood. To improve the efficiency of model training, we utilize the observation of the canvas $c_{t}^{x,\sigma }$, which depends only on the first *t* elements of σ. Hence we can combine into one pass the loss calculations of trajectories that are the same in the first *t* steps but different at the *t* + 1 step. Switching the summation order of σ and *t*, we have Equation (2). It leads to an efficient training algorithm: sample *t* from 0 to *n* − 1 and partial permutation σ_1: *t*_, construct the canvas $c_{t}^{x,\sigma }$. Therefore, the loss is calculated as Equation (3).^[^ ^53^ ^]^

(1) $$\begin{array}{ccc} \log p(x;\theta ) & = & \log\sum_{\sigma \in S_{n}}\prod_{t=0}^{n-1}p\left(a_{t}^{x,\sigma }|c_{t}^{x,\sigma };\theta \right)\geq \log\left(n!\right)\\ & & +\;\frac{1}{n!}\sum_{\sigma \in S_{n}}\sum_{t=0}^{n-1}\log p\left(a_{t}^{x,\sigma }|c_{t}^{x,\sigma };\theta \right) \end{array}$$

where equality holds when the posterior *p*(σ|*x*; θ) is uniform. By maximizing this lower bound, we do not favor any particular order, but encourage the model to realize *x* equally well in all orders. It can help the model to complete any partial input text regardless of the position of the blanks.

(2) $$\begin{array}{cc} & \sum_{t=0}^{n-1}\frac{1}{n!}\sum_{\sigma \in S_{n}}\log p\left(a_{t}^{x,\sigma }|c_{t}^{x,\sigma };\theta \right)\\ & \;=n\cdot E_{t}E_{\sigma_{1:t}}E_{\sigma_{t+1}}E_{\sigma_{t+2:n}}\left[\log p\left(a_{t}^{x,\sigma }|c_{t}^{x,\sigma };\theta \right)\right]\\ & \;=n\cdot E_{t}E_{\sigma_{1:t}}E_{\sigma_{t+1}}\left[\log p\left(a_{t}^{x,\sigma }|c_{t}^{x,\sigma };\theta \right)\right]\\ & \;=E_{t}E_{\sigma_{1:t}}\left[\frac{n}{n-t}\sum_{\sigma_{t+1}}\log p\left(a_{t}^{x,\sigma }|c_{t}^{x,\sigma };\theta \right)\right] \end{array}$$

(3) $$\;-\log\left(n!\right)-\frac{n}{n-t}\sum_{\sigma_{t+1}}\log p\left(a_{t}^{x,\sigma }|c_{t}^{x,\sigma };\theta \right)$$

where the θ is the network weights; $c_{t}^{x,\sigma }$ is the *t*th canvas with the specified training formula *x* and the selected generation order (permutation σ); $a_{t}^{x,\sigma }$ is the action at time *t* which includes the blank selection, element picking, and blank option selection.

In our BLMM model, we use the 118 elements plus seven special tokens including <PAD>,<UNK>,<FIRST>,<LAST>,<EOS>,<BLANK>,<BLANK_0> as the vocabulary for training the BLMM models. If some elements are too infrequent, the model can remove those elements from the vocabulary. The network model parameters are specified in the hyper‐parameter part of the Experimental Section.

### 2.3 De Novo Generative Design of Materials Composition

#### 2.3.1 Generation of Hypothetical Materials Compositions:

We prepare two sets of training datasets as described in the Experimental Section to train different BLMM models for materials composition generation. The first set includes three datasets ICSD‐mix, MP‐mix, OQMD‐mix, with selected compositions from Inorganic Crystal Structure Database (ICSD),^[^ ^67^ ^]^ Materials Project (MP),^[^ ^6^ ^]^ and Open Quantum Materials Database (OQMD) databases^[^ ^7^ ^]^ respectively, all of which include samples that do not satisfy charge neutrality or balanced electronegativity. The second set of datasets includes ICSD‐pure, MP‐pure, OQMD‐pure, which only includes selected formulas that are charge‐neutral with balanced electronegativity. For each of these datasets, we train a BLMM transformer model and use it to generate 100 000 hypothetical formulas.

During the generation process, the model uses two different strategies in the generation process: greedy sampling and beam search sampling. Greedy sampling selects the markers with the highest probability at each step and always takes the action with the highest probability. On the other hand, beam search sampling explores multiple candidate sequences in parallel and selects the most promising ones based on their cumulative probabilities. When decoding with beam search, we used beam sizes of 1, 5, and 10, and choose the best value as observed on the validation set. Additionally, the beam search in the BLMM does not search for the sentence with the maximum marginal likelihood *p*(*x*; θ), but rather for a sentence and a trajectory that have the maximum joint likelihood *p*(*x*, σ; θ). Given its ability to generate high‐quality and diverse outputs by simultaneously considering multiple potential sequences, we adopt the beam search sampling approach in our work.

**Figure 2:**
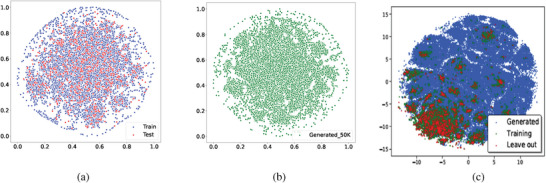
Distribution of existing materials and hypothetical materials generated by our BLMM‐Pure‐ICSD model. The distributions are generated by calculating the one‐hot representation for the compositions and then using T‐Sne to project them into two‐dimension space. a) Distribution of known training‐testing formulas. b) Distribution of generated samples by BLMM. c) Distribution of training‐testing and generated samples of MATGAN (from ref. [12]). Note that (a) and (b) are embedded using a single t‐sne run.

To evaluate whether our language BLMM models can learn the chemistry of inorganic materials (compositions) and use it to generate valid hypothetical formulas, we first check the distribution of the generated samples with respect to the training set and holdout test set of the Pure‐ICSD dataset. We first represent each formula using the one‐hot encoding as described in ref. [12] and then map all the sample matrix representations into two‐dimension space using the t‐SNE algorithm. The results are shown in **Figure** **2**. First, we find that the compositions of existing materials in the ICSD dataset are not evenly distributed, but grouped into several clusters corresponding to materials families (Figure 2a). We then find that the known materials (training and testing samples) are only a tiny portion of the whole composition space and our generated samples are almost evenly mixed with training and testing samples, indicating that our generators can greatly expand the chemical compositions in the chemical design space. To show the overlap of generated samples with the training set, Figure 2b shows only the generated samples without the training and test samples. We can find that in the center of the space, a large number of generated samples are close to training and testing samples. Compared to the distribution of generated samples (Figure 2c) by MATGAN in ref. [12], our generated samples show much higher similarity with known materials.

For composition generators, it is interesting to check how the generated compositions are distributed within known families of compounds, which is how materials researchers understand composition generations. Here, we have collected all the 6296 nitrides, 21 344 oxides, and 1063 ABC_3_ compounds from the ICSD‐pure training set. Similarly, we collect 96 277 nitrides, 26 9547 oxides, and 7008 ABC compositions from the 482 055 generated compositions by our BLMM model trained with the ICSD‐pure training set. We then calculate the Magpie features of these compositions and do tsne‐embedding to map three pairs of compositions separately, namely nitrides of training and generated samples, oxides of training and generated samples, and ABC_3_ compositions of training and generated ones. The final distributions of these three families of compositions are shown in Figure S4 (Supporting Information), in which the blue dots are training samples while the green dots are generated compositions by BLMM. We find that for both nitrides and oxides, the BLMM generates not only the samples that are closely mixed with the training samples, but also compositions that go beyond the regions of the training compositions, indicating its capability to generate non‐trivial variation of known compositions.

#### 2.3.2 Evaluation of BLMM Generation Performance using Validity, Uniqueness, Recovery Rate, and Novelty

We evaluate the performance of our BLMM generators and compare it with that of the baseline random formula generator and the GAN‐based MATGAN using four evaluation criteria, including recovery rate, validity (including charge‐neutrality and electronegativity balance), uniqueness, and novelty as described in the Experimental Section. It should be noted that while these multiple criteria can all reflect the generation performance of the generator models, they are not all equal. Actually, only the recovery rate directly measures the capability of the models for generating compositions that can form stable structures, which makes it the most important criterion compared to others.

We train six different models using six different datasets, as shown in Table 5 of the Experimental Section and then evaluate their generation performance.

**Table 2:** Emergence of orders within the generated samples over training epochs. In the beginning, the sequence of element symbols is mostly random. As the training process goes on, the elements in the generated samples are more ordered by their electronegativity as the training samples show.

Epoch	Sample	Formula
1	F I As O Rb O O K F F	KRbAsI(OF)_3_
5	O O Na S Rh O O O O O	NaRhSO_7_
10	Cd Mo O Mo O Mo O O	CdMo_3_O_4_
15	Cr O F O O F	CrO_3_F_2_
20	H H N O Cl O O	H_2_NClO_3_
25	Ta Ta Se N Se O O O O	Ta_2_Se_2_NO_4_
30	Ba Ba Ge F O F F F O	Ba_2_Ge(OF_2_)_2_
50	Ba Ba Fe Fe O Cl O O	Ba_2_Fe_2_ClO_3_
100	Sm Sm Sm Ni O O O O O O	Sm_3_NiO_6_
150	Li Li Ti Zn Zn O O O O O	Li_2_TiZn_2_O_5_
200	Sr Bi Br O O O O O	SrBiBrO_5_

**Table 3:** BLMM for element substitution and material doping.

SrTiO_3_	SrTiO_3_	LiFePO_4_	Li_2_MnO_3_	Sr_3_GaN_3_
Sr_OOO	_Ti OOO	Li_POOOO	Li Li _ OOO	SrSrSr_NNN
Ti 0.051	Ba 0.286	Co 0.206	O 0.098	Sr 0.082
Ru 0.046	Ca 0.209	Mn 0.150	Li 0.056	B 0.074
Si 0.044	Sr 0.144	Fe 0.129	C 0.056	Ir 0.046
Ge 0.038	Mg 0.089	Cu 0.120	Si 0.045	Fe 0.043
C 0.036	Ti 0.045	Ni 0.117	Mn 0.040	Ga 0.042
Sn 0.034	Y 0.036	Cr 0.070	Co 0.038	Cr 0.042
Mn 0.031	Na 0.032	V 0.033	Cr 0.030	N 0.040
O 0.028	K 0.030	Zn 0.026	Fe 0.029	Co 0.040
Ir 0.028	Li 0.029	Mg 0.023	Ti 0.028	Ge 0.033
Zr 0.027	Rb 0.021	Li 0.021	O 0.028	Mn 0.033

**Table 4:** The predicted thermodynamically stable binary and ternary materials with zero energy above the hull and negative formation energy (*E*_form_). The mp‐id of the template structure of each material is also mentioned.

Formula	mp‐id of template	$E_{\mathrm{form}}$ [eV]
CrH_3_	mp‐557539	–1.3496
Cu_2_N	mp‐1077346	–1.4302
IrN_3_	mp‐1252369	–2.1322
MoN_2_	mp‐754064	–3.9658
NbCl_5_	mp‐1095321	–2.4986
IrN_2_	mp‐1019082	–2.8743
RuN_2_	mp‐1101173	–3.3132
Cr_2_S_3_	mp‐2750	–0.6802
GeSe_2_	mp‐1095294	–0.2522
Rb_3_TlF_6_	mp‐24034	–2.3948
MnNiN_2_	mp‐1226009	–2.9510
Rb_2_MnF_5_	mp‐1213714	–2.6915

**Table 5:** Six datasets used in experiments: pure datasets only include selected samples with a neutral charge and balanced electronegativity; mixed datasets do not have such limits. The test sets are only used in computing recovery rates for evaluation.

Mix datasets				Pure datasets			
	ICSD‐mix	OQMD‐mix	MP‐mix		ICSD‐pure	OQMD‐pure	MP‐pure
Total	52317	363182	89121	Total	39431	216540	63703
Train	50,755	345022	84664	Train	37459	205713	60517
Valid	1336	9080	2228	Valid	986	5413	1593
Test	1,336	9080	2228	Test	986	5413	1593

**Figure 3:**
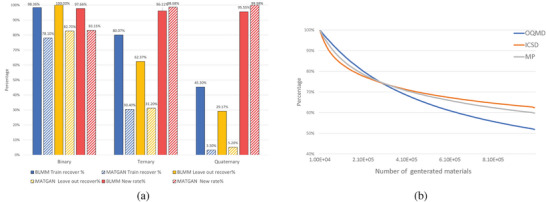
Recovery rate, novelty, and uniqueness of the BLMM generator trained with ICSD‐pure compared to the baseline MATGAN. a) Distribution of the recovery rates of the training and validation/testing samples as well as the novelty: the percentages of newly generated hypothetical materials out of all generated samples. For all binary, ternary, and quaternary materials, BLMM shows much higher recovery rates for both the training set and the holdout set. b) Comparison of the uniqueness curves of the generated samples by BLMM trained with different datasets.

We first check our BLMM models' capability for generating new material compositions that can form stable structures, which can be measured by the recovery rate of known compound compositions. Compared to the rates of novelty, charge‐neutrality and balanced electronegativity, the recovery rate is the most important metric for evaluating composition generators. We use the BLMM model trained with the ICSD‐pure dataset to generate 1 million compositions and obtain 784,829 binary/ternary/quaternary compositions, which are then used to calculate the recovery rates and novelty. First, we check whether our BLMM models can learn the chemical composition rules of the training set by calculating the training set recovery rates. The results of our BLMM model are shown in **Figure** **3**a solid blue bars. Our model has recovered as much as 98.36% of the 2624 binary compounds in the training set due to the limited combinations of binary compositions. The model also recovers 80.07% ternary compounds and 45.3% quaternary compounds in the training set. These recovery rates are all significantly higher than those of the baseline MATGAN models (blue striped bars) reported in ref. [12] including 78.1% for binary, 30.4% for ternary, and 3.3% for quaternary compounds. This significantly higher recovery rate of BLMM over the training samples (2.63 and 13.7 times of MATGAN for ternary and quaternary compounds, respectively) indicates our BLMM model's capability to learn known chemical composition distribution.

The recovery rates of our BLMM model on the hold‐out dataset are shown as solid orange bars in Figure 3. Our model achieves recovery percentages of 100% of 59 binary materials, 62.37% of 372 ternary materials, and 29.17% of 360 quaternary ones. The reason that the quaternary compounds have a much lower recovery rate is that the quaternary design space is much higher.^[^ ^68^ ^]^ Since these holdout ICSD samples are all experimentally synthesized materials that are not contained in the training set, the high holdout recovery rates of BLMM directly demonstrate our model's capability to generate chemically valid real material compositions. Considering the huge quaternary compound space ( 4.1 × 10^12^), the 29.17% recovery rate of our BLMM model to find 105 samples out of 360 samples within only 1 million generated samples is a feat comparable to finding a needle in a haystack. In contrast, the holdout recovery rates of MATGAN for binary, ternary, and quaternary compounds are only 82.7%, 31.2%, and 5.2%. Our BLMM model's holdout recovery rates over the quaternary holdout samples is 5.6 times of MATGAN.

We also check the novelty of our BLMM model, which measures the percentage of the generated samples that are not within the known ICSD dataset. Our model achieves 97.66%, 96.11%, and 95.55% for binary, ternary and quaternary compounds respectively, indicating its strong capability to explore new materials.

Another important performance measure of composition generators is the uniqueness, which calculates the percentage of unique samples out of all generated samples.^[^ ^69^ ^]^ Here for the three BLMM models trained with OQMD‐pure, MP‐pure, and ICSD‐pure datasets, we calculate the uniqueness percentages at the end of every 10,000 generated samples up to 1 million. The results are shown in Figure 3. First, we find that all three models have shown high uniqueness: even after generating one million samples, the uniqueness percentages remain above 50%: OQMD (51.87%), ICSD (62.38%) and MP (59.73%). The difference of these three models may be attributed to their different distributions of the training set. The OQMD dataset is mainly composed of ternary materials (>84.4%)^[^ ^12^ ^]^ so the BLMM–OQMD‐pure model tends to generate ternary samples, while the total number of chemically valid ternary compounds with integer ratios as estimated by SMACT (Semiconducting Materials from Analogy and Chemical Theory) to be around 200 000. So, it tends to generate more duplicate ternary samples. In contrast, the ratio of binary/ternary/quaternary is about 1:5.3:4.7 for the ICSD‐pure dataset. For MP‐pure dataset, it is about 1:7.3:6, which is much more balanced than the previous two. It is interesting to find that before generating about 300 000 samples, the ICSD has the smallest uniqueness while OQMD has the highest one. The probable reason is that the OQMD dataset used here is much larger (216 540 samples) compared to MP‐pure (63 703 samples) and ICSD‐pure (39 431 samples), which covers more combinations of elements allowing it to generate diverse formulas in the beginning. However, after the inflection point at around 300 000 generated samples, the BLMM–OQMD model has visited most of the ternary formulas which it prefers to generate, causing it to fail to continue to generate new formulas, leading to the lowest uniqueness. In contrast, the BLMM–ICSD is trained with more balanced binary, ternary, and quaternary compounds, enabling it has the capability to generate diverse compositions even after 300 000 samplings. Compared to the MATGAN generators in ref. [12], we find that the uniqueness of our BLMM–OQMD is higher that of GAN–OQMD while the BLMM–ICSD has a similar uniqueness of 75% as their GAN–ICSD. However, their GAN–ICSD has a much higher uniqueness of around 87% than the 75% of our BLMM–ICSD. This is likely due to that our BLMM is a probabilistic model that uses neural networks to explicitly learn the context dependency among the elements within the compositions, which makes it tend to more closely approximate the elemental combinations. Instead, the GAN model used in MATGAN implicitly learns to approximate the distributions of training set as determined by the discriminator model, which makes them have fewer constraints that allow them to explore the chemical design space more freely.

**Figure 4:**
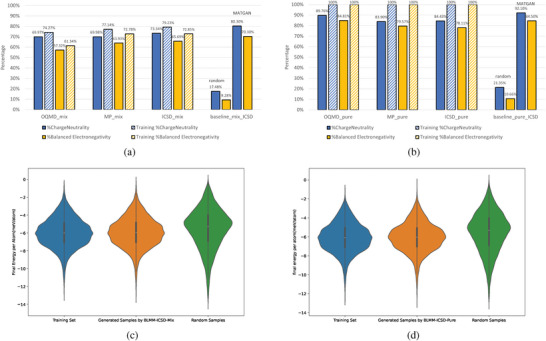
Validity performance of BLMM composition generators. a) The percentages of charge‐neutral (CN) and electronegativity‐balanced (EN) samples out of all generated samples by the BLMM models trained with mixed samples compared to those of the baseline random composition generator and MATGAN. b) The percentage of charge‐neutral (CN) and electronegativity‐balanced (EN) samples out of all generated samples by the BLMM models trained with CN and EN samples compared to those of the random composition generator and MATGAN. c) The formation energy per atom distribution of random samples, training set, and generated samples for BLMM–ICSD‐mix models in (a). d) The formation energy per atom distribution of random samples, training set, and generated samples for BLMM–ICSD‐pure models in (c).

We further check the validity performance of three BLMM models trained with datasets OQMD‐mix, MP‐mix, and ICSD‐mix as shown in **Figure** **4**a. The OQMD‐mix training dataset contains 345022 material compositions, with 74.27% samples satisfying charge neutrality (CN) and 61.34% samples are electronegativity‐balanced (EN). Out of the 100 000 generated samples, we find that up to 69.97% satisfy charge neutrality and 57.32% meet balanced electronegativity, two of the major chemical validity requirements, indicating that our BLMM model has learned the chemical rules for assembling chemically valid compositions. The MP‐mix training set has higher percentages in terms of charge neutrality and balanced electronegativity compared to OQMD‐mix. It contains, however, only 84 664 samples. However, our BLMM models do not suffer from this significantly smaller dataset and still achieve high percentages of charge neutrality (69.98%) and balanced electronegativity (63.93%) for the 100 000 generated samples. While both OQMD‐mix and MP‐mix contain computationally derived materials (mainly via element substitutions), the ICSD‐mix training set contains only 50 755 experimentally synthesized materials. Our BLMM model trained with this dataset shows even higher percentages of charge neutrality (73.34%) and balanced electronegativity (65.69%) for the 100 000 generated samples. As a comparison, we use the random composition generator (see Experimental Section) to generate 100 000 compositions using the anonymous formulas of the BLMM‐ICSD‐mix generated samples, which helps it avoid the issue of generating too random invalid formulas. Even with this lifting, the samples generated by the random generator only contain only 17.48% of samples with charge neutrality and 9.28% samples with balanced electronegativity. It is thus shown that our BLMM model achieves more than four and six times better performance in terms of generating chemically valid materials compositions compared to the random generator. As noted in the Evaluation Criteria section, the charge‐neutrality performance of our BLMM is actually under‐estimated. A re‐evaluation of charge‐neutrality using the Pymatgen with its fractional oxidation states, the CN percentage of the BLMM–ICSD‐mix generated samples reaches 87.99% while the training set CN percentage reaches 88.93%.

It is well‐known that both the MP and OQMD databases contain a large number of materials with high e‐above‐hull energy that may not be stable or synthesizable. To check their influence on the generation performance, we select the formulas with e‐above‐hull energy less than 0.05 eV from the Material Project dataset for BLMM model training. We found that 85.43% of the generated 69 519 formulas satisfy charge‐neutrality and 82.12% satisfy charge‐neutrality and electronegativity balance requirements, respectively, which are both much higher than those generated with all samples without using e‐hull‐energy standard (69.98% and 63.93%) for data selection. This verifies that stricter data selection can train BLMM models to generate better quality compositions.

We realize that the validity performance of our generators depends on the validity level of the training sets. To check if better training sets can improve the validity, we re‐trained three models using the OQMD‐pure, MP‐pure, and ICSD‐pure datasets, which all contain only samples that satisfy charge‐neutrality and balanced electronegativity. The results are shown in Figure 4b. For the BLMM model trained with OQMD‐pure, it now achieves charge neutrality of 89.76% compared to 69.97% of the model trained with OQMD‐mix, a significant 19.78% improvement despite its 40% smaller dataset size (205 713 vs 345 022), indicating the importance of data quality versus quantity for our BLMM model. For the BLMM models trained with MP‐pure and ICSD‐pure, a similar significant validity performance is observed: the BLMM–MP‐pure's charge neutrality percentage has been improved by 13.92% and balanced electronegativity percentage by 15.64%. For the BLMM–ICSD‐pure model, these two validity performances have also been improved by 11.09% and 12.42%. In comparison, the lifted random generator only achieves charge‐neutrality of 21.35% and balanced electronegativity of 10.66% for their 100 000 generated samples.

We also compare the validity performance of our BLMM models with our previously developed MATGAN models that are based on generative adversarial networks.^[^ ^12^ ^]^ We find the MATGAN model trained with ICSD‐mix achieves 80.3% CN and 70.3% EN compared to our 73.34% CN and 65.69% EN, with about a 4–6% advantage. Their GAN model trained with ICSD‐pure achieves 92.1% CN and 84.5% EN compared to BLMM's 84.43% and 78.11%, also a 6–7% advantage. However, our hyper‐parameter tuning experiments have shown that our BLMM models' performance can be further improved. The main interesting fact here is that, as shown in Figure 2, our BLMM models are complementary to the MATGAN models: BLMM tends to generate hypothetical materials similar to the training samples, good for tinkering, while MATGAN models are good for exploring new compositions.

Another way to check the generation performance is to evaluate the stability of the generated compositions by predicting their formation energy. We first use the BLMM model trained with the ICSD‐mix dataset to generate 100 000 samples and after filtering, 83 465 hypothetical samples remain. We then use the Roost‐based formation energy predictor (see Experimental Section) trained with all the Materials Project dataset to predict their formation energy. We also use the anonymous composition templates of these generated samples to create 83 465 random samples and predict their formation energy. The formation energy distributions of the ICSD‐mix training set, the generated samples and the random samples are shown in Figure 4c. We find that the formation energy distribution of our BLMM‐generated samples is much more similar to the training set compared to that of the random samples which have much more samples with formation energy closer to zero or above zero. We find the predicted formation energies of most of the generated samples are lower than 0 eV, indicating their potential dynamic stability. Figure 4d shows the similar formation energy distributions for the training, generated, and random samples of the BLMM–ICSD‐pure model, which contains 635 051 generated and random samples respectively.

#### 2.3.3 Process of BLMM's Learning of Chemical Rules:

**Figure 5:**
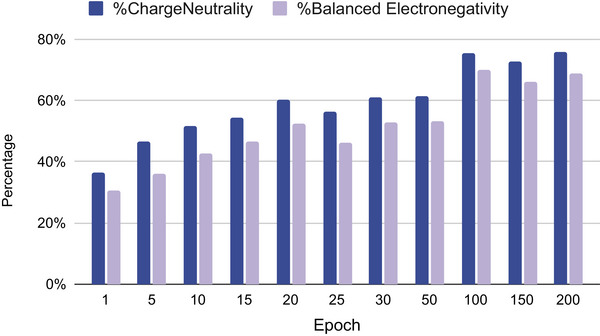
Increasing percentages of charge‐neutral and electronegativity balanced samples generated by the BLMM models saved over the training process. In the beginning, few samples satisfy these two chemical validity rules. As the training goes on, the models gradually gain the capability to generate chemically valid materials compositions.

To illustrate the chemical order/rules that emerge during the training process of our BLMM model, we save the intermediate models at the end of 1/5/10/15/20/25/30/50/100/150/200 epochs of training using the MP‐mix dataset. We then generate 10 000 samples for each of these models and calculate the percentages of charge‐neutral samples and electronegativity balanced samples. The results are shown in **Figure** **5**. We find that in the beginning, only a low percentage of the generated samples satisfy these two basic chemical rules: less than 20% when the models are trained with less than 10 epochs. However, when the training epochs surpass 25 epochs, the percentage of charge‐neutral samples has already reached more than 50% while the percentage of balanced electronegativity is slightly lower. When the training epochs reach 200, the %chargeNeutrality has already reached almost 70% and %balanced electronegativity reaches 64%.

To get an intuitive understanding of orders shown in the generated samples, **Table** **2** shows the typical generated oxide samples for the models saved at the end of the different epochs. At epoch 1, the elements of the composition are almost randomly ordered. When the models are trained with 5–50 epochs, the formulas already include both anions and cations for creating charge‐neutral compositions. However, the elements of the same type are still not completely ordered as in the training samples. Even though formally, the appearance order of the elements in the formula does not make chemical difference, it is a violation of the orders within the training samples. When the training epochs reach 50, we find almost all the generated samples have the correct element order: elements of the same type are shown together forming different clusters and the oxygen appears at the end of the formulas just as the training samples. This indicates that our BLMM model has learned the implicit chemical rules/order from our training set. Table S1 (Supporting Information) shows more generated samples by the models saved at epochs ranging from 1 to 200.

### 2.4 Tinkering Design of Materials Using Blank Filling Transformer Networks

#### 2.4.1 Design of New Materials Using BLMM

One of the major advantages of BLMM language model‐based composition generator compared to GAN‐based models^[^ ^12^ ^]^ is that it allows conditional composition generation starting with the templates from known crystal materials, by which we can specify some elements prior, and then the model will fill the remaining blanks. This can be very useful for exploratory materials search. To demonstrate this capability, we start with Perovskite SrTiO_3_. We mask Ti from the expanded formula sequence Sr _ O O O, and feed it to our BLMM model trained with the MP‐mix dataset, the model suggests a list of possible filling elements as shown in **Table** **3** column 1 (only the top 20 suggestions are shown here). The element with the highest ranking is Ti with a probability score of 0.051. The other suggested elements include Ru, Si, Ge, C, Sn, Mn, Ir, Pt, Cr, Mo, which all lead to valid material entries included in the Materials Project database. We then mask the Sr element from SrTiO_3_ and feed it to the network, the model suggests the Sr as the third best candidate. The other suggestions include Ba, Ca, Mg, Cs, Zr which all form valid entries in the Materials Project database. Three of the suggestions including Li, Rb, Ta are not in the database. We check the charge neutrality and electronegativity balance for these three hypothetical compositions LiTiO_3_, RbTiO_3_, TaTiO_3_ using the SMACT package as implemented at MaterialsAtlas.org website.^[^ ^70^ ^]^ They all satisfy these two chemical rules, indicating their potential to be valid crystals.

We further mask the Fe element from the lithium‐ion battery cathode material LiFePO_4_ and feed it to our model, which suggests Mn and Co with probabilities of 0.206 and 0.15, leading to LiMnPO_4_ and LiCoPO_4_, two candidate cathode materials under study.^[^ ^71^ ^]^ We find the probabilistic BLMM model is much more flexible compared to the GAN model for composition generation.^[^ ^12^ ^]^ For example, our model can be used to find doping elements for tuning crystal properties. We mask the Mn element in the Li_2_MnO_3_, a well‐known ionic conductor and feed it to the network model, which suggests Co, Cr, Ni, Ti, Fe, all have been used as doping elements in experimental studies.^[^ ^72^, ^73^ ^]^ To further prove our model can discover new materials, we mask the Ga element in a known material Sr_3_GaN_3_, which is not contained in the training set for our model. We feed the masked sequence Sr Sr Sr _ O O O to our model, which not only identifies the masked element Ga but also suggests Cr as a substitution element, which leads to the rediscovery of an important electrode material Sr_3_CrN_3_ as studied in ref. [74] and another known crystal Sr_3_FeN_3_.^[^ ^75^ ^]^ Please note that despite demonstrating the potential of our BLMM model for dopant element suggestion in tinkering materials, its performance has yet to be quantitatively evaluated.

#### 2.4.2 BLMM Learns Materials Chemistry

To evaluate whether our BLMM model learns the implicit chemical rules for composing feasible materials, we select a dataset of compositions that are charge‐neutral, have balanced electronegativity and unique oxidation state assignments as estimated by the Pymatgen oxidation guess module. The last requirement makes it nontrivial to select appropriate elements for substitution. We also require the maximum number of atoms for each element to be less or equal to 10 for fast oxidation states calculation. In total, we obtain 47 737 material compositions. We then expand each of the formulas (e.g., SrTiO_3_ –> Sr Ti O O O) and randomly mask one element in the sequence and run the blank filling using our BLMM model. We then check the charge neutrality and electronegativity balance after element substitution compared to the performance by random element substitution. Our experiment shows that BLMM can achieve 92.6% charge neutrality and 90.8% with balanced electronegativity after BLMM suggested missing element filling. By comparison, the random element substitution can succeed in 89.1% for charge neutrality and 80.5% for balanced electronegativity, indicating that our BLMM models have successfully learned the chemical rules of inorganic material compositions. It should be noted that the surprising random substitution's 80.5% is due to the replacement of a single atom over a charge‐balanced formula with balanced electronegativity.

To further understand how our probabilistic transformer BLMM model works in generating chemically valid material compositions, we analyze the oxidation state patterns of those elements recommended by BLMM for filling the blank of _TiO_3_. Since the oxidation states of SrTiO_3_ is Sr(+2)Ti(+4)O(–2)_3_, we are expecting an element with +2 oxidation state. Table S2 (left half) (Supporting Information) shows the top 15 elements recommended by BLMM. Top six elements (Ba, Ca, Sr, Mg, Ti, Y) all have +2 as one of their accessible oxidation states(including Sr). The top four elements (Ba, Ca, Sr, Mg) are all from the same element group. The 12th–15th recommended elements (Ta, Zr, Sc, Hf) also have +2 as the accessible oxidation states. It is interesting to see that the 7th–11th recommended elements do not have +2 as their accessible oxidation states. A close examination of Table S2 (left, gray part) (Supporting Information) shows that these five elements all have +1 as their common accessible oxidation state. The reason is that for SrTiO_3_, there are three possible accessible OS assignments that can make the formula charge‐neutral: Sr(+2)Ti(+4)O(–2)_3_, Sr(+2)Ti(+1)O(–1)_3_, Sr(+1)Ti(+2)O(–1)_3_. The masked element can take either +2 or +1 to satisfy the charge‐neutrality requirement (since O(–1) is unusual, the recommended elements with +1 OS are ranked much lower than most elements with +2 OS). Note that this requirement is not fed into or built‐into our BLMM model: it is an implicitly learned rule by BLMM as it is trained with many charge‐neutral material compositions. This demonstrates the huge potential for a BLMM‐like deep learning language model to uncover hidden chemical or physical rules from raw data. We did a similar analysis for the tinkering design of Sr_3GaN_3_ by masking Ga element with oxidation state +3 and asks BLMM for suggestions (See Table S2 (right half), Supporting Information). We find that out of the top 15 suggestions, 13 of them have +3 as one of their accessible oxidation states (the other two are Sr elements aiming to switch Ga to Sr). We also find that the ranked elements after the 15th element Hf all have low probability scores and all do not have +3 as accessible OS, which indicates that our BLMM model has intrinsically learned that the oxidation states of elements determine the charge‐neutrality of the compositions.

### 2.5 Conditional Generative Design of Materials with High Bandgap

**Figure 6:**
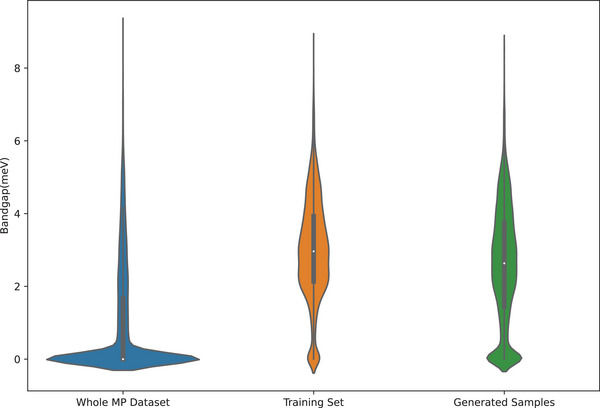
Bandgap distribution for a) the whole Materials Project materials; b) the training set of high‐bandgap materials for the BLMM model; c) the generated samples by BLMM. The bandgap distribution of the generated ones is much closer to the training set than the whole dataset.

To evaluate whether our language model can capture the composition patterns for high‐bandgap materials, we collect 29 772 formulas with bandgap above 1.98 eV from the Materials Project (for those formulas with multiple phases, we include it if it has one phase with a bandgap greater than 2.0 eV). We then trained the BLMM language generator and used it to generate 100 000 formulas. We then use the composition‐based bandgap prediction model (See Experimental Section) to predict the bandgaps of these hypothetical materials and plot their distribution against the bandgap distribution of the training set. As shown in **Figure** **6**, the bandgap distribution of our hypothetical compositions is much closer to the training set compared to the bandgap distribution of all materials project samples, which indicates that the BLMM‐bandgap model has learned the implicit rules to generate high‐bandgap materials.

### 2.6 New Materials Predicted by Our Algorithm and Validated Using DFT

**Figure 7:**
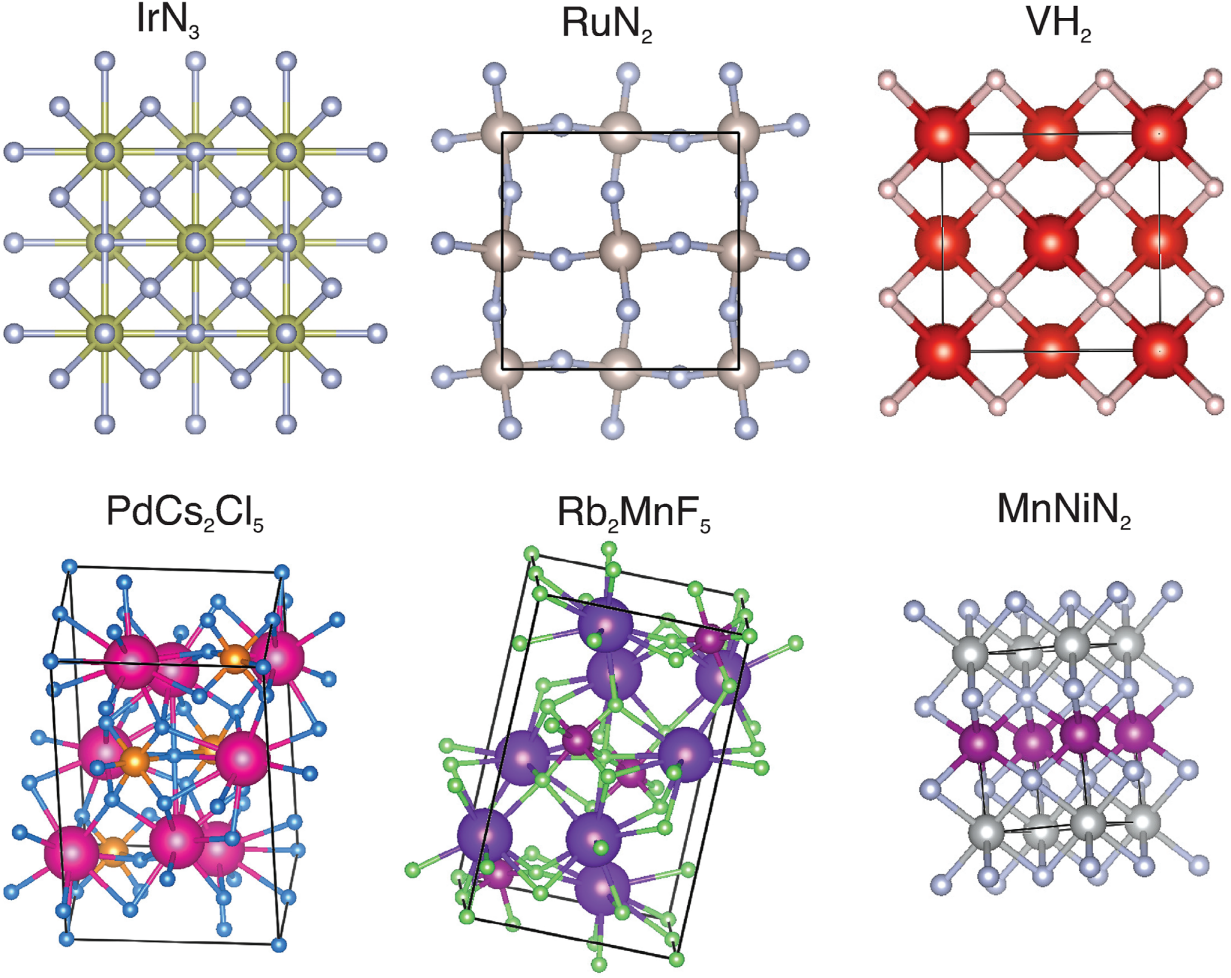
Candidate structures with zero e‐above‐hull energy discovered by our BLMM model (Supporting Information contains all the 13 structures of Table 4).

Due to the difficulty of DFT simulations with compounds with La and Ar family elements, we prepare a subset of ICSD compositions that excludes those elements and use it to train a BLMM model (Detailed hyper‐parameters are described in the Supporting Information). We generate 100 000 ternary and quaternary material compositions and then we predict their formation energy using the composition‐based formation energy prediction model (See Experimental Section). Next, we calculate their total energy and predict their e‐above‐hull energies to rank these candidates. We then pick the top 100 formulas with the lowest predicted e‐above‐hull energy and apply our TCSP, a template‐based crystal structure prediction algorithm^[^ ^18^ ^]^ to obtain the structures. For the predicted structures with the best quality scores, we run DFT relaxation to get the final structures and calculate their formation energy and e‐above‐hull using VASP (see Experimental Section). **Table** **4** shows the 14 discovered binary and ternary materials along with their formation energy. The crystal structures of three binary and three ternary materials are presented in **Figure** **7** with more structures shown in Figures S2– S4 (Supporting Information). All the cif files of these 14 newly discovered DFT‐validated crystal materials are appended to the Supporting Information.

## 3 Discussion

We developed a transformer‐based blank filling language model for the generative design of materials compositions. The large‐scale experiments on both composition generation and tinkering/element substitution show that they have learned strong chemical rules for creating chemically valid compositions. Especially, compared with previous GAN‐based generators,^[^ ^12^ ^]^ our probabilistic BLMM model brings much higher explainability of the tinkering suggestions and more control of the generation process of new compositions, which are highly desirable for materials scientists.

By comparing the distribution of generated samples versus the training samples (Figure 2) of our BLMM model compared to those by the MATGAN model (Figure 3 of ref. [12]), we find our generated samples share a much higher similarity in its composition distribution compared to that of the training samples while the GAN based generator tends to create very different composition families, indicating that BLMM models are more suitable for the exploitation of known chemical space while MATGAN models are more suitable for exploring new chemical design space. This major difference may come from their very different modeling and learning mechanisms: BLMM models explicitly learn the chemical context dependency (element dependency within known formulas) while the MATGAN models lack such explicit probabilistic modeling components and rely on the neural network models to indirectly approximate their composition to fool the discriminator, leading to less control of the generation process. Another major difference is that our BLMM models are much slower than MATGAN when generating compositions. In Table S1 (Supporting Information), we summarize the major performance and technical differences of BLMM and the baseline MATGAN models for material composition generation. We also compared our BLMM with the Crystal Diffusion Variational Autoencoder (CDVAE) model,^[^ ^76^ ^]^ which specializes in structure generation. Using CDVAE, we generated 8 090 unique composition structures and computed their charge‐neutral (CN), electronegativity‐balanced (EN), and combined (CNEN) metrics. CDVAE achieved 87.44% for CN, and 78.29% for both EN and CNEN. In comparison, our BLMM model trained on the MP dataset achieved 83.90% for CN, and 79.57% for both EN and CNEN. While CDVAE slightly outperforms BLMM in charge neutrality, likely due to its incorporation of structural information, BLMM shows a marginal advantage in electronegativity balance. Importantly, as a generative model, CDVAE has limitations in generating high‐symmetry structures, particularly those with space group numbers above 100. BLMM, focusing solely on composition, doesn't face such structural generation constraints and can potentially cover a wider range of material compositions, including those with high symmetry when paired with advanced structure prediction methods. This makes BLMM particularly valuable for rapid exploration of compositional space, especially when structural data is limited or unavailable. BLMM's focus on composition allows it to potentially capture a broader range of materials, including those with high symmetry that CDVAE might struggle to generate.

To provide a broader perspective, we expanded our experiments by merging the Materials Project and GNoME datasets^[^ ^77^ ^]^ to obtain approximately 80 000 samples for both model training and validation. Among the 89 546 unduplicated results generated, the best‐performing model trained on CNEN (pure) data excluding the Lanthanide family demonstrated consistent or slightly improved results compared to other datasets. Specifically, the model achieved 92.8% for CN and 89.82% for both EN and CNEN. This consistent or enhanced performance on larger and more diverse datasets underscores the potential of our model for real‐world materials discovery applications. This improved performance indicates the robustness and applicability of our model across different datasets, reinforcing its utility in practical settings. The ability to maintain high performance on larger and more varied datasets highlights the scalability and effectiveness of our approach, making it a promising tool for advancing materials discovery.

For decades, chemists and materials scientists have relied on heuristic knowledge or some chemical rules to explore the chemical design space and find new materials. These rules can be described using certain chemical grammars as suggested in ref. [30], which can significantly reduce the sampling errors compared to random composition generation while exhaustive enumeration and screening is infeasible since the number of quaternary compounds can already exceed 10^12^. While a few heuristic grammar rules can be deduced by human experts, there is a huge risk of missing many unknown grammar rules or some implicit grammar rules not analytically expressible by the grammar. In this regard, our deep transformer network models have big advantages. First, our model implicitly uses the data‐driven strategy to learn the composition generation grammars from the known materials composition data, which avoids the pitfalls of human‐defined chemical grammars. Our BLMM models demonstrate up to eight times improvement when generating charge neutral and electronegativity balanced materials compositions compared to the baseline random generator as shown in Figure 4b. In addition, the probabilistic generative process of our model shares the advantages of the grammar rules in terms of their interpretability as it tells which elements are most suitable given a specific chemical composition context. We would like to note that our transformer models learn the charge‐neutrality rules of material compositions without requiring a specific order of the elements. Our initial experiments used elements sorted by electronegativity, but additional experiments with shuffled element orders provided compelling evidence that our model learns underlying compositional rules rather than relying on specific element positions. In these new experiments, we trained the model on a dataset where the element orders were randomized. The generated samples mirrored these shuffled orders, but when converted back to ordered sequences, the charge neutrality (CN), balanced electronegativity (EN), and their combination (CNEN) scores remained consistent with the original ordered training set (84.8%, 77.6%, and 77.6% respectively for 9722 samples). This robustness to input order is a key advantage of our approach, allowing it to generalize across various representation schemes. It demonstrates that our model effectively learns the underlying materials “charge neutrality grammar” from the data, regardless of the input sequence order. However, we found that when using sorted element token sequences by electronegativity, the model learns to generate sequences with the same sorting order. These findings further underscore the flexibility and power of our deep transformer network approach in capturing complex chemical relationships without relying on predetermined rules or specific input formats.

While our models can generate chemically valid hypothetical materials compositions in terms of charge neutrality and electronegativity balance, their synthesizability and stability remain uncertain without crystal structures. Recent advancements in crystal structure prediction, including template‐based,^[^ ^18^, ^19^ ^]^ deep learning‐based,^[^ ^78^ ^]^ and global optimization‐based approaches,^[^ ^17^, ^79^ ^]^ have expanded our ability to predict structures for various material families. These tools can be integrated with our BLMM composition generators, which offer significant advantages in interpretability and control, crucial for materials scientists in the initial composition determination phase. Looking forward, incorporating more advanced crystal structure prediction algorithms and formation energy prediction models (replacing our current TCSP pipeline and ROOST, respectively) could further enhance the validation process. This integration of composition generation with advanced structure prediction and property calculation tools presents a promising direction for future research, potentially bridging the gap between hypothetical compositions and practical materials development.

## 4 Experimental Section

### Dataset

To evaluate the performance of the language model‐based generator, two sets of models were trained with two different types of datasets. The first category of datasets were all screened formulas from ICSD/MP/OQMD with the number of elements less than nine, the number of atoms in a unit cell less than 100, and without fractional coordinates. These datasets might contain a certain amount of materials that were not charge‐neutral or balanced electronegativity. The second category of datasets were those samples from the first category but were charge‐neutral and had balanced electronegativity. The details of these datasets are shown in **Table** **5**.

### Baseline Pseudo Random Composition Generator

‐pseudo random composition generator was created as the baseline. For all generated samples, the numbers of samples were counted with a different number of elements from 2 to *E*_*n*_. Then for each of the element number *K*, the same number of composition samples with *K* elements were generated. For each of them, an atom number from 1 to 20 was randomly selected for each of the *K* elements. This would ensure the distribution of binary, ternary, etc. samples was the same as the comparison group.

### DFT Calculations

To check the structural stability of the predicted materials, the first‐principles calculations based on the density functional theory (DFT) were applied using the Vienna ab initio simulation package (VASP).^[^ ^80^, ^81^, ^82^, ^83^ ^]^ The projected augmented wave (PAW) pseudopotentials, where 520 eV plane‐wave cutoff energy, were employed to treat the electron‐ion interactions.^[^ ^84^, ^85^ ^]^ The exchange‐correlation functional was considered with the generalized gradient approximation (GGA) based on the Perdew–Burke–Ernzerhof (PBE) method.^[^ ^86^, ^87^ ^]^ The energy convergence criterion was set as 10^−5^ eV. The atomic positions were optimized with the force convergence criterion of 10^−2^ eV Å^−1^. The Brillouin zone integration for the unit cells was determined by employing the Γ‐centered Monkhorst–Pack *k*‐meshes. The Formation energies (in eV per atom) of the materials were estimated based on the formula in Equation (4), where *E*[Material] is the total energy per unit formula of the target structure, *E*[A_*i*_] is the energy of *i* ^th^ element of the material, *x*_*i*_ indicates the number of A_*i*_ atoms in a unit formula, and *n* is the total number of atoms in a unit formula(*n* = ∑_*i*_ *x*_*i*_). The energy above the hulls of the materials with negative formation energies were calculated using the Pymatgen code.^[^ ^88^ ^]^

(4) $$E_{\mathrm{form}}=\frac{1}{n}\left(E\left[\mathrm{Material}\right]-\sum_{i}x_{i}E\left[A_{i}\right]\right)$$

### Evaluation Criteria

The performance of materials generative models could be mainly evaluated using three criteria including validity, uniqueness, and recovery rate.^[^ ^12^ ^]^ Here the validity of generated samples was evaluated using the charge neutrality and electronegativity balance, which are two fundamental chemical rules of crystals. The SMACT's charge‐neutrality and electronegativity balance check module was used for validation,^[^ ^89^ ^]^ which had the following assumptions: 1) it assumes each element in a composition/formula can only take one integer oxidation state; 2) the charge‐neutrality check results depend on which oxidation states for each element is used (e.g. Pymatgen or ICSD oxidation table); 3) the module cannot reliably check the CN of intermetallic compounds since they do not follow the usual valence rules. These assumptions explained why the ICSD‐mix training set only had 72.89% charge‐neutrality. When fractional oxidation states were allowed in charge‐neutrality check as done by the Pymatgen,^[^ ^88^ ^]^ the CN percentage reached 88.93%, which however assumes the existence of multiple oxidation states for single elements. Since in the absence of structural information for such an assumption, the default SMACT setting for charge‐neutrality check was chosen, which may under‐estimate the CN performance of the BLMM models.

It was interesting to check how the generated samples from the BLMM models satisfy these rules without explicit enforcement of such rules during model training. To do this, the charge‐neutrality and electronegativity check procedure as proposed in ref. [89] was adopted to calculate the percentages of samples that obey these rules within the training and generated sets.

The uniqueness of a generative model measured by the percentage of the number of unique samples out of the number of all generated samples (n). The higher this measure, the better the capability of the model could generate diverse samples.

The recovery rate measured the percentage of samples from the training or testing set that have been regenerated by the generator model. The high recovery rate over the test set indicated that a generator has a high discovery performance since the test set samples were known crystals that actually exist. A related measure was the novelty of a generator, which measures the percentage of the generated samples that are new samples (do not exist before).

### Hyper‐Parameters

Hyper‐parameter studies of the BLMM model were conducted using the ICSD‐pure dataset to evaluate how the major parameters affect the model performance. A set of BLMM models was trained with a maximum number of epochs of 3000 and different hyper‐parameter configurations. To evaluate their composition generation performance, each of these models was used to generate 10 000 formulas and calculate the percentages of charge‐neutral (CN) and electronegativity‐balanced (EN) samples out of all the generated samples. The hyper‐parameters evaluated here include the number of transformer layers, the number of transformer heads, the size of the hidden layers, dropout rate and learning rate. Instead of an exhaustive enumeration of all possible parameter configurations, a default hyper‐parameter set was used which includes six transformer layers, eight transformer heads, and the size of the hidden layer was 2048. The default the dropout rate and the learning rate were 0.3 and 0.0001. Then each time, one hyper‐parameter was changed and their CN/EN percentages were calculated. In addition, the performance of two other related language models LBLM and INST^[^ ^53^ ^]^ was also compared using the same default hyper‐parameter set.

**Figure 8:**
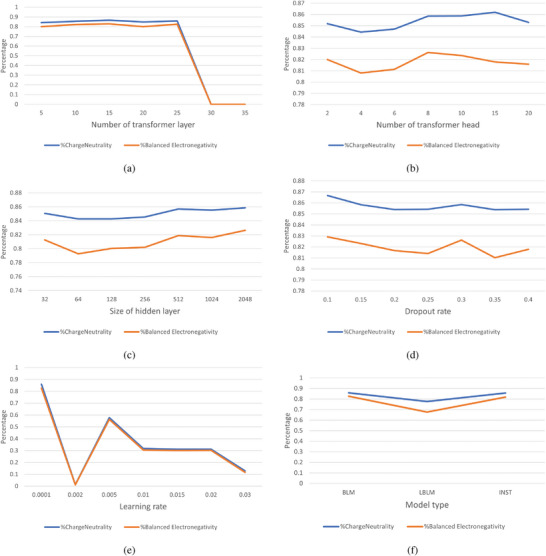
Hyper‐parameter tuning of BLMM materials composition generator. a) The percentages of charge‐neutral (CN) and electronegativity‐balanced (EN) samples out of all generated samples by the BLMM models trained with different numbers of the transformer layers. b) The CN/EN percentages of the models trained with different numbers of the transformer heads. c) The CN/EN percentages of the models trained with different sizes of the hidden layer. d) The CN/EN percentages of the BLMM models trained with different dropout rate. e) The CN/EN percentages of the BLMM models trained with different learning rates. f) The CN/EN percentages of the BLMM models compared to the LBLM model and INST model.

How the number of transformer layers affects the CN/EN percentages of generated samples was initially checked. As shown in **Figure** **8**a, these trained models achieve good performance when the number of transformer layers ranges from 5 to 25 without significant differences. The models achieved the charge‐neutrality (CN) percentages of 84.26%, 85.56%, 86.64%, 0.8494%, 85.91% and EN percentages of 79.97%, 82.11%, 82.88%, 80.02%, 82.48% for for 5/10/15/20/25 transformer layers, respectively. However, when the number of transformer layers reached 30 and 35, these models could not generate a contiguous sequence of elements but inserted a < *blank* > between every two elements in the generated sequence, leading to invalid formulas.

Second, how the number of transformer heads affects the BLMM model performance was evaluated. Figure 8c shows that the model had a good performance in terms of CN/EN percentages when the number of transformer heads is eight. When the number of transformer heads reached 15, the model had the best CN percentage of 86.19%, though it was not a significant improvement from the performance of the model with eight transformer heads.

Next, experiments were conducted with different sizes of the inner hidden layers ranging from 32 to 2048. The hidden layer sizes were varied from 32 to 2048 (the default size of the hidden layer). Figure 8b shows that the models with larger sizes of hidden layers (512/1024/2048) achieved better performance compared to the models with smaller hidden layer sizes. The model performance changes with different dropout rates ranging from 0.1 to 0.4 were further evaluated. In Figure 8d, the models had good performance when the dropout rates were 0.1 and 0.3. The impact of the learning rate over generation performance is shown in Figure 8e. It was found that the model performs well with CN/EN percentages of 85.85% and 82.63% respectively only when the learning rate is 0.0001 (the default learning rate). The maximum number of epochs were determined to run for the training by plotting the training and validation loss curves. It was found that for most of the model training, the losses converge within 3000 epochs. So 3000 was chosen as the no. of epochs to train the models.

Finally, the performance of BLMM was compared with two other related language models: LBLM and INST.^[^ ^53^ ^]^ As shown in Figure 8f, BLMM achieved the best performance in both charge‐neutrality and balanced electronegativity (EN) percentages, with 85.85% and 82.63% respectively. The INST model also had good performance with CN/EN percentages of 85.69% and 81.76% respectively. The default hyper‐parameter setting is shown in the Supporting Information.

### Formation Energy and Bandgap Prediction Models Based on Roost

To check the quality of generated compositions, composition‐based prediction models were trained for both formation energy and bandgaps using the dataset downloaded from the Materials Project database.^[^ ^6^ ^]^ The machine learning model used was roost, a graph message passing neural network as described in ref. [90]. The training set of Roost‐FE contained 98 290 unique compositions which were downloaded from the Materials Project database. All compositions with multiple phases would only keep the lowest formation energy records. The Roost–Bandgap model was trained with 113 501 samples. The formation energy roost model achieved a validation MAE of 0.113 eV while the bandgap predictor achieved an MAE of 0.236 eV as evaluated on the 5% hold‐out test sets.

### Web App for Materials Design

**Figure 9:**
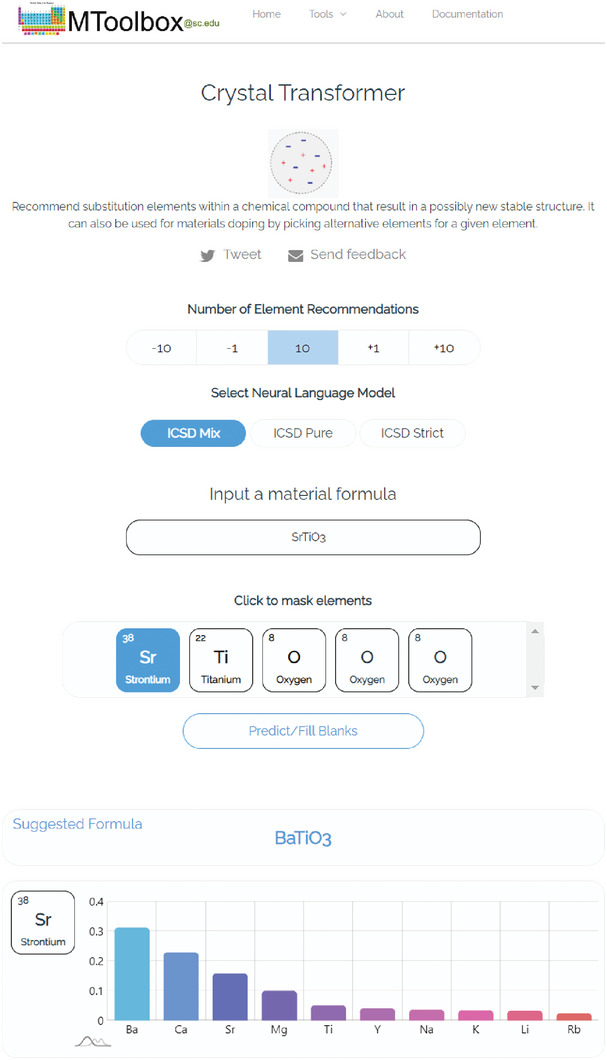
The Crystal Transformer web app facilitates the design of new stable structures by recommending element substitutions. Users can input a material formula and receive instant suggestions, as demonstrated here with SrTiO_3_, for which the app proposes BaTiO_3_ as a potential stable variant.

To facilitate the design and tinkering of materials, a user‐friendly web app available at www.materialsatlas.org/blmtinker was developed. This innovative tool addresses a critical need in the materials science community for accessible, AI‐driven resources that can accelerate the discovery and optimization of novel materials. The web app leverages the advanced neural language models to provide real‐time suggestions for material design, significantly reducing the time and resources typically required for exploratory research. By making advanced materials informatics tools widely accessible, this platform has the potential to catalyze breakthroughs across diverse fields, including energy storage and conversion, electronics, and structural materials. **Figure** **9** illustrates the interface of the Crystal Transformer web app, which aids in designing materials by recommending element substitutions within a chemical compound to potentially create new stable structures. Users can input a material formula, select a neural language model, and adjust the number of element recommendations. For instance, when SrTiO_3_ is entered and the ICSD‐MIX model is selected, the app suggests BaTiO_3_ as a recommendation, accompanied by a bar graph displaying the probabilities of various element substitutions. Furthermore, the web app includes comprehensive documentation, encompassing usage guidelines and technical specifications.

## Conflict of Interest

The authors declare no conflict of interest.

## Author Contributions

J.H. led the conceptualization and supervision of the project, and was responsible for funding acquisition. The methodology was developed by J.H., L.W., Q.L., D.S., Y.S., and R.D. Software development was carried out by J.H., L.W., S.S., Y.S., and N.F. J.H. provided the resources for the study. The original draft preparation was undertaken by J.H., L.W., and E.S., with subsequent review and editing by J.H., L.W., D.S., and F.C. Visualization was handled by L.W., Y.S., Q.L., and J.H.

## Supporting information

Supporting Information

## Data Availability

The data that support the findings of this study are openly available in Materials Project at https://www.materialsproject.org, reference number 1. These data were derived from the following resources available in the public domain: [MaterialsProject], https://www.materialsproject.org; [OQMD], https://www.oqmd.org.

## References

[1] J. E. Saal , A. O. Oliynyk , B. Meredig , Annu. Rev. Mater. Res. 2020, 50, 49.

[2] Y. Liu , O. C. Esan , Z. Pan , L. An , Energy and AI 2021, 3, 100049.

[3] A. Zunger , O. I. Malyi , Chem. Rev. 2021, 121, 3031. 33481581 10.1021/acs.chemrev.0c00608

[4] A. A. Emery , J. E. Saal , S. Kirklin , V. I. Hegde , C. Wolverton , Chem. Mater. 2016, 28, 5621.

[5] S. Curtarolo , G. L. Hart , M. B. Nardelli , N. Mingo , S. Sanvito , O. Levy , Nat. Mater. 2013, 12, 191. 23422720 10.1038/nmat3568

[6] A. Jain , S. P. Ong , G. Hautier , W. Chen , W. D. Richards , S. Dacek , S. Cholia , D. Gunter , D. Skinner , G. Ceder , K. A. Persson , APL Mater. 2013, 1, 1.

[7] S. Kirklin , J. E. Saal , B. Meredig , A. Thompson , J. W. Doak , M. Aykol , S. Rühl , C. Wolverton , npj Comput. Mater. 2015, 1, 1.

[8] S. Curtarolo , W. Setyawan , S. Wang , J. Xue , K. Yang , R. H. Taylor , L. J. Nelson , G. L. Hart , S. Sanvito , M. Buongiorno‐Nardelli , N. Mingo , O. Levy , Comput. Mater. Sci. 2012, 58, 227.

[9] W. Zhao , J. Ding , Y. Zou , C.‐a. Di , D. Zhu , Chem. Soc. Rev. 2020, 49, 7210. 32975251 10.1039/d0cs00204f

[10] L. Ye , X. Hu , X. Wang , F. Chen , D. Tang , D. Dong , K. Xie , J. Mater. Chem. A 2019, 7, 2764.

[11] J. Zhou , L. Shen , M. D. Costa , K. A. Persson , S. P. Ong , P. Huck , Y. Lu , X. Ma , Y. Chen , H. Tang , Y. P. Feng , Scientific Data 2019, 6, 1. 31189922 10.1038/s41597-019-0097-3 PMC6561947

[12] Y. Dan , Y. Zhao , X. Li , S. Li , M. Hu , J. Hu , npj Comput. Mater. 2020, 6, 1.

[13] Y. Sawada , K. Morikawa , M. Fujii , arXiv preprint arXiv:1910.11499 2019.

[14] J. Noh , J. Kim , H. S. Stein , B. Sanchez‐Lengeling , J. M. Gregoire , A. Aspuru‐Guzik , Y. Jung , Matter 2019, 1, 1370.

[15] T. Xie , J. C. Grossman , Phys. Rev. Lett. 2018, 120, 145301. 29694125 10.1103/PhysRevLett.120.145301

[16] K. T. Schütt , H. E. Sauceda , P.‐J. Kindermans , A. Tkatchenko , K.‐R. Müller , J. Chem. Phys. 2018, 148, 24. 10.1063/1.5019779 29960322

[17] A. Oganov , A. Lyakhov , M. Valle , G. Frapper , in CECAM‐Workshop Lausanne, Lausanne, Switzerland 2012, pp. 22–26.

[18] L. Wei , N. Fu , E. Siriwardane , W. Yang , S. S. Omee , R. Dong , R. Xin , J. Hu , Inorg. Chem. 2021. 10.1021/acs.inorgchem.1c03879 35420427

[19] M. Kusaba , C. Liu , R. Yoshida , arXiv preprint arXiv:2201.11188 2022.

[20] D. Hicks , M. J. Mehl , E. Gossett , C. Toher , O. Levy , R. M. Hanson , G. Hart , S. Curtarolo , Comput. Mater. Sci. 2019, 161, S1.

[21] S. D. Griesemer , L. Ward , C. Wolverton , Phys. Rev. Mater. 2021, 5, 105003.

[22] B. Zheng , J. Fan , B. Chen , X. Qin , J. Wang , F. Wang , R. Deng , X. Liu , Chem. Rev. 2022, 122, 5519. 34989556 10.1021/acs.chemrev.1c00644

[23] X. Ma , L. Yang , K. Lei , S. Zheng , C. Chen , H. Song , Nano Energy 2020, 78, 105354.

[24] E. Bustarret , C. Marcenat , P. Achatz , J. Kačmarčik , F. Lévy , A. Huxley , L. Ortéga , E. Bourgeois , X. Blase , D. Débarre , J. Boulmer , Nature 2006, 444, 465. 17122852 10.1038/nature05340

[25] G. Xiao , Q. Liu , S. Wang , V. G. Komvokis , M. D. Amiridis , A. Heyden , S. Ma , F. Chen , J. Power Sources 2012, 202, 63.

[26] M. Li , Y. Wang , Y. Wang , F. Chen , C. Xia , ACS Appl. Mater. Interfaces 2014, 6, 11286. 24971668 10.1021/am5017045

[27] Z. Wang , Y. Liu , T. Sato , M. Hohenadler , C. Wang , W. Guo , F. F. Assaad , Phys. Rev. Lett. 2021, 126, 205701. 34110204 10.1103/PhysRevLett.126.205701

[28] D. Gunning , M. Stefik , J. Choi , T. Miller , S. Stumpf , G.‐Z. Yang , Sci. Rob. 2019, 4, 7120. 10.1126/scirobotics.aay7120 33137719

[29] W. Samek , G. Montavon , S. Lapuschkin , C. J. Anders , K.‐R. Müller , Proc. IEEE 2021, 109, 247.

[30] J. T. Margraf , Z. W. Ulissi , Y. Jung , K. Reuter , ChemRxiv . 2021.

[31] J. Wei , D. Garrette , T. Linzen , E. Pavlick , in *Proc. 2021 Conf. Emp. Meth. Nat. Lang. Proc*., Association for Computational Linguistics, Online and Punta Cana, Dominican Republic, 2021, pp. 932–948.

[32] D. Silver , T. Hubert , J. Schrittwieser , I. Antonoglou , M. Lai , A. Guez , M. Lanctot , L. Sifre , D. Kumaran , T. Graepel , T. Lillicrap , K. Simonyan , D. Hassabis , Science 2018, 362, 1140. 30523106 10.1126/science.aar6404

[33] J. Devlin , M.‐W. Chang , K. Lee , K. Toutanova , arXiv preprint arXiv:1810.04805 2018.

[34] J. Achiam , S. Adler , S. Agarwal , L. Ahmad , I. Akkaya , F. L. Aleman , D. Almeida , J. Altenschmidt , S. Altman , S. Anadkat , R. Avila , I. Babuschkin , S. Balaji , V. Balcom , P. Baltescu , H. Bao , M. Bavarian , J. Belgum , I. Bello , J. Berdine , G. Bernadett‐Shapiro , C. Berner , L. Bogdonoff , O. Boiko , M. Boyd , A.‐L. Brakman , G. Brockman , T. Brooks , M. Brundage , K. Button , et al., arXiv preprint arXiv:2303.08774 2023.

[35] S. Rothe , S. Narayan , A. Severyn , Trans. Assoc. Comput. Linguist. 2020, 8, 264.

[36] J. Li , T. Tang , W. X. Zhao , J.‐R. Wen , arXiv preprint arXiv:2105.10311 2021.

[37] N. Brandes , D. Ofer , Y. Peleg , N. Rappoport , M. Linial , Bioinformatics 2022, 38, 2102. 35020807 10.1093/bioinformatics/btac020 PMC9386727

[38] A. Madani , B. McCann , N. Naik , N. S. Keskar , N. Anand , R. R. Eguchi , P.‐S. Huang , R. Socher , Progen: Language modeling for protein generation, *arXiv preprint arXiv:2004.03497* 2020.

[39] L. Yu , Y. Su , Y. Liu , X. Zeng , Brief. Funct. Genom. 2021, 20, 323. 10.1093/bfgp/elab036 34342611

[40] X.‐C. Zhang , C.‐K. Wu , Z.‐J. Yang , Z.‐X. Wu , J.‐C. Yi , C.‐Y. Hsieh , T.‐J. Hou , D.‐S. Cao , Brief. Bioinform. 2021, 22, 152. 10.1093/bib/bbab152 33951729

[41] P. Schwaller , T. Laino , T. Gaudin , P. Bolgar , C. A. Hunter , C. Bekas , A. A. Lee , ACS Cent. Sci. 2019, 5, 1572. 31572784 10.1021/acscentsci.9b00576 PMC6764164

[42] H. Kim , J. Na , W. B. Lee , J. Chem. Inf. Model. 2021, 61, 5804. 34855384 10.1021/acs.jcim.1c01289

[43] V. Bagal , R. Aggarwal , P. Vinod , U. D. Priyakumar , J. Chem. Inf. Model. 2021, 62, 2064. 34694798 10.1021/acs.jcim.1c00600

[44] D. Rothchild , A. Tamkin , J. Yu , U. Misra , J. Gonzalez , arXiv preprint arXiv:2108.10307 2021.

[45] L. Wei , N. Fu , Y. Song , Q. Wang , J. Hu , J. Cheminform. 2023, 15, 88. 37749655 10.1186/s13321-023-00759-z PMC10518939

[46] E. C. Alley , G. Khimulya , S. Biswas , M. AlQuraishi , G. M. Church , Nat. Methods 2019, 16, 1315. 31636460 10.1038/s41592-019-0598-1 PMC7067682

[47] O. Dollar , N. Joshi , D. A. Beck , J. Pfaendtner , Chem. Sci. 2021, 12, 8362. 34221317 10.1039/d1sc01050f PMC8221056

[48] V. Tshitoyan , J. Dagdelen , L. Weston , A. Dunn , Z. Rong , O. Kononova , K. A. Persson , G. Ceder , A. Jain , Nature 2019, 571, 95. 31270483 10.1038/s41586-019-1335-8

[49] L. Antunes , K. Butler , R. Grau‐Crespo , arXiv:2307.04340 2023.

[50] T. Gupta , M. Zaki , N. A. Anoop Krishnan , Mausam , npj Comput. Mater. 2022, 8, 102.

[51] J. Dagdelen , A. Dunn , S. Lee , N. Walker , A. S. Rosen , G. Ceder , K. A. Persson , A. Jain , Nat. Commun. 2024, 15, 1418. 38360817 10.1038/s41467-024-45563-x PMC10869356

[52] Q. Zhou , P. Tang , S. Liu , J. Pan , Q. Yan , S.‐C. Zhang , Proc. Natl. Acad. Sci. 2018, 115, E6411. 29946023 10.1073/pnas.1801181115 PMC6048531

[53] T. Shen , V. Quach , R. Barzilay , T. Jaakkola , in *Proc. 2020 Conf. Emp. Meth. Nat. Lang. Proc. (EMNLP)*, Association for Computational Linguistics, Online, 2020, pp. 5186–5198.

[54] C. W. Glass , A. R. Oganov , N. Hansen , Comput. Phys. Commun. 2006, 175, 713.

[55] Y. Wang , J. Lv , L. Zhu , Y. Ma , Comput. Phys. Commun. 2012, 183, 2063.

[56] Y. Wang , J. Lv , L. Zhu , S. Lu , K. Yin , Q. Li , H. Wang , L. Zhang , Y. Ma , J. Phys.: Condens. Matter 2015, 27, 203203. 25921406 10.1088/0953-8984/27/20/203203

[57] S. S. Omee , L. Wei , M. Hu , J. Hu , J. Mater. Inform. 2024, 4.

[58] G. Hautier , C. Fischer , V. Ehrlacher , A. Jain , G. Ceder , Inorg. Chem. 2011, 50, 656. 21142147 10.1021/ic102031h

[59] W. Sun , C. J. Bartel , E. Arca , S. R. Bauers , B. Matthews , B. Orvañanos , B.‐R. Chen , M. F. Toney , L. T. Schelhas , W. Tumas , J. Tate , A. Zakutayev , S. Lany , A. M. Holder , G. A. Ceder , Nat. Mater. 2019, 18, 732. 31209391 10.1038/s41563-019-0396-2

[60] T. Lookman , P. V. Balachandran , D. Xue , R. Yuan , npj Comput. Mater. 2019, 5, 21.

[61] J. Schmidt , M. R. Marques , S. Botti , M. A. Marques , npj Comput. Mater. 2019, 5, 1.

[62] K. T. Butler , D. W. Davies , H. Cartwright , O. Isayev , A. Walsh , Nature 2018, 559, 547. 30046072 10.1038/s41586-018-0337-2

[63] P. Raccuglia , K. C. Elbert , P. D. Adler , C. Falk , M. B. Wenny , A. Mollo , M. Zeller , S. A. Friedler , J. Schrier , A. J. Norquist , Nature 2016, 533, 73. 27147027 10.1038/nature17439

[64] G. Schwank , K. Basler , Cold Spring Harbor Perspect. Biol. 2010, 2, 001669. 10.1101/cshperspect.a001669 PMC2827898 20182606

[65] Z. Fan , J. Hu , K. Seo , E. Goodman , R. Rosenberg , B. Zhang , in *2001 Genetic and Evolutionary Computation Conference Late Breaking Papers*, San Francisco, California, USA, 2001, pp. 81–86.

[66] Z. Yang , Z. Dai , Y. Yang , J. Carbonell , R. R. Salakhutdinov , Q. V. Le , Adv. Neural Inform. Proc. Syst. 2019, 32.

[67] A. Belsky , M. Hellenbrandt , V. L. Karen , P. Luksch , Acta Crystallogr. Sect. B: Struct. Sci. 2002, 58, 364. 10.1107/s0108768102006948 12037357

[68] D. W. Davies , K. T. Butler , A. J. Jackson , A. Morris , J. M. Frost , J. M. Skelton , A. Walsh , Chem 2016, 1, 617. 27790643 10.1016/j.chempr.2016.09.010 PMC5074417

[69] D. Polykovskiy , A. Zhebrak , B. Sanchez‐Lengeling , S. Golovanov , O. Tatanov , S. Belyaev , R. Kurbanov , A. Artamonov , V. Aladinskiy , M. Veselov , A. Kadurin , S. Johansson , H. Chen , S. Nikolenko , A. Aspuru‐Guzik , A. Zhavoronkov , Front. Pharmacol. 2020, 11, 1931. 10.3389/fphar.2020.565644 PMC7775580 33390943

[70] J. Hu , S. Stefanov , Y. Song , S. S. Omee , S.‐Y. Louis , E. Siriwardane , Y. Zhao , L. Wei , npj Comput. Mater. 2022, 8, 1.

[71] N. Nitta , F. Wu , J. T. Lee , G. Yushin , Mater. Today 2015, 18, 252.

[72] G. Singh , R. Thomas , A. Kumar , R. Katiyar , J. Electrochem. Soc. 2012, 159, A410.

[73] Q. Fu , F. Du , X. Bian , Y. Wang , X. Yan , Y. Zhang , K. Zhu , G. Chen , C. Wang , Y. Wei , J. Mater. Chem. A 2014, 2, 7555.

[74] P. Chanhom , K. E. Fritz , L. A. Burton , J. Kloppenburg , Y. Filinchuk , A. Senyshyn , M. Wang , Z. Feng , N. Insin , J. Suntivich , G. Hautier , J. Am. Chem. Soc. 2019, 141, 10595. 31251610 10.1021/jacs.9b03472

[75] S. Kikkawa , T. Yamamoto , K. Ohta , M. Takahashi , F. Kanamaru , The Chemistry of Transition Metal Carbides and Nitrides, Chapman and Hall, USA 1996, pp. 175–190.

[76] T. Xie , X. Fu , O.‐E. Ganea , R. Barzilay , T. Jaakkola , arXiv preprint arXiv:2110.06197 2021.

[77] A. Merchant , S. Batzner , S. S. Schoenholz , M. Aykol , G. Cheon , E. D. Cubuk , Nature 2023. 10.1038/s41586-023-06735-9 PMC10700131 38030720

[78] J. Hu , Y. Zhao , Y. Song , R. Dong , W. Yang , Y. Li , E. Siriwardane , arXiv preprint arXiv:2102.01620 2021.

[79] X. Shao , J. Lv , P. Liu , S. Shao , P. Gao , H. Liu , Y. Wang , Y. Ma , J. Chem. Phys. 2022, 156, 014105. 34998332 10.1063/5.0074677

[80] G. Kresse , J. Hafner , Phys. Rev. B 1993, 47, 558. 10.1103/physrevb.47.558 10004490

[81] G. Kresse , J. Hafner , Phys. Rev. B 1994, 49, 14251. 10.1103/physrevb.49.14251 10010505

[82] J. F. G. Kresse , Comput. Mater. Sci. 1996, 6, 15.

[83] G. Kresse , J. Furthmüller , Phys. Rev. B 1996, 54, 11169. 10.1103/physrevb.54.11169 9984901

[84] P. E. Blöchl , Phys. Rev. B 1994, 50, 17953. 10.1103/physrevb.50.17953 9976227

[85] G. Kresse , D. Joubert , Phys. Rev. B 1999, 59, 1758.

[86] J. P. Perdew , K. Burke , M. Ernzerhof , Phys. Rev. Lett. 1996, 77, 3865. 10062328 10.1103/PhysRevLett.77.3865

[87] J. P. Perdew , K. Burke , M. Ernzerhof , Phys. Rev. Lett. 1997, 78, 1396. 10.1103/PhysRevLett.77.3865 10062328

[88] S. P. Ong , W. D. Richards , A. Jain , G. Hautier , M. Kocher , S. Cholia , D. Gunter , V. L. Chevrier , K. A. Persson , G. Ceder , Comput. Mater. Sci. 2013, 68, 314.

[89] D. W. Davies , K. T. Butler , A. J. Jackson , J. M. Skelton , K. Morita , A. Walsh , J. Open Source Softw. 2019, 4, 1361.

[90] R. E. Goodall , A. A. Lee , Nat. Commun. 2020, 11, 1. 33293567 10.1038/s41467-020-19964-7 PMC7722901

