# Supplementary material for: Crystal Composition Transformer: Self‐Learning Neural Language Model for Generative and Tinkering Design of Materials
Source: Adv Sci (Weinh). 2024 Aug 5;11(36):2304305. doi: 10.1002/advs.202304305 (PMC11423232; doi:10.1002/advs.202304305)
Supplement: Supplementary file 1 — Supporting Information [file ADVS-11-2304305-s001.pdf]

ADVANCED SCIENCE
Open Access

Supporting Information

for *Adv. Sci.*, DOI 10.1002/advs.202304305

Crystal Composition Transformer: Self-Learning Neural Language Model for Generative and Tinkering Design of Materials

*Lai Wei, Qinyang Li, Yuqi Song, Stanislav Stefanov, Rongzhi Dong, Nihang Fu, Edirisuriya M. D. Siriwardane, Fanglin Chen and Jianjun Hu**

Supplemental Information

Crystal Composition Transformer: Self-Learning Neural Language Model for Generative and Tinkering Design of Materials

Lai Wei, Qinyang Li, Yuqi Song, Stanislav Stefanov, Rongzhi Dong, Nihang Fu, Edirisuriya M. D. Siriwardane, Fanglin Chen, Jianjun Hu*

Default Hyperparameters for Model Training

We list the training parameters of our BLMM network. These settings were used to achieve the results presented in Table 2 of the main text.

d_inner_hid: 2048
n_head: 8
n_layers: 6
dropout rate: 0.3
epoch: 3000
lr: 0.0001

Summary of the comparisons of BLMM and MATGAN

We provide a detailed comparison between our proposed BLMM model and the baseline MATGAN model, highlighting the key differences in performance and technical aspects.

Table S1. Performance and technical comparison of BLMM and MATGAN (baseline)

	MATGAN	BLMM (this paper)
Performance of recovery rate	31.20% for ternary<br>5.2% for quaternary	62.37% for ternary<br>29.17% for quaternary
Composition representation	One-hot matrix	Sequence of tokens
Neural network structure	Convolutional network	Probabilistic Transformer network
Generation process	Uncontrollable	Controllable
Tinkering design	Not possible	Possible (works well)
Web app	None	ttp://materialsatlas.org/blmtinker

Oxidation state analysis of BLMM mechanism

We present an analysis of the oxidation states for elements suggested by the BLMM. We examine two specific cases: (a) filling the blank in $\_TiO_3$ (derived from $SrTiO_3$) and (b) filling the blank in $Sr_3\_N_3$ (derived from Sr_3GaN_3). This analysis provides insights into the model's understanding of chemical composition and its ability to suggest chemically plausible elements.

Table S2. Oxidation states of suggested elements by BLMM for filling (a) $\_TiO_3$. (b) $Sr_3\_N_3$

$\_TiO_3$ (from $SrTiO_3$)				$Sr_3\_N_3$ (from Sr_3GaN_3)		
Sr [+2]		[1, 2]		Ga [+3]		[-5, -4, -3, -2, -1, 0, 1, 2, 3]
Element	Probability	Element oxidation states		Element	Probability	Element oxidation states
Ba	0.286	[2]		Sr	0.08	[1, 2]
Ca	0.209	[1, 2]		B	0.07	[1, 2, 3]
Sr	0.144	[1, 2]		Ir	0.05	[-3, -1, 1, 2, 3, 4, 5, 6, 7, 8]
Mg	0.09	[1, 2]		Fe	0.04	[-2, -1, 1, 2, 3, 4, 5, 6]
Ti	0.045	[-1, 1, 2, 3, 4]		Ga	0.04	[1, 2, 3]
Y	0.036	[1, 2, 3]		Cr	0.04	[-2, -1, 1, 2, 3, 4, 5, 6]
Na	0.032	[-1, 1]		N	0.04	[-3, -2, -1, 1, 2, 3, 4, 5]
K	0.03	[-1, 1]		Co	0.04	[-1, 1, 2, 3, 4, 5]
Li	0.029	[1]		Ge	0.03	[-4, -3, -2, -1, 1, 2, 3, 4]
Rb	0.021	[-1, 1]		Mn	0.03	[-3, -2, -1, 1, 2, 3, 4, 5, 6, 7]
Cs	0.016	[-1, 1]		Al	0.03	[1, 2, 3]
Ta	0.007	[-1, 1, 2, 3, 4, 5]		Sr	0.03	[1, 2]
Zr	0.006	[1, 2, 3, 4]		Ru	0.02	[-2, 1, 2, 3, 4, 5, 6, 7, 8]
Sc	0.006	[1, 2, 3]		C	0.02	[-4, -3, -2, -1, 1, 2, 3, 4]
Hf	0.006	[2, 3, 4]		Ni	0.02	[-1, 1, 2, 3, 4]

Supplemental figures

We present additional figures that show ternary materials discovered by our BLMM. Figure S1-S3 display structures predicted using the generated compositions and the TCSP template-based crystal structure prediction algorithm. As shown in Figure S4, we compare the distribution of materials generated by BLMM to those in its training dataset. This comparison focuses on nitrides, oxides, and ABC_3 compounds.

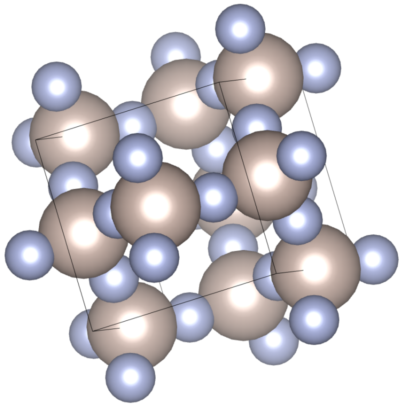

(a) RuN_2

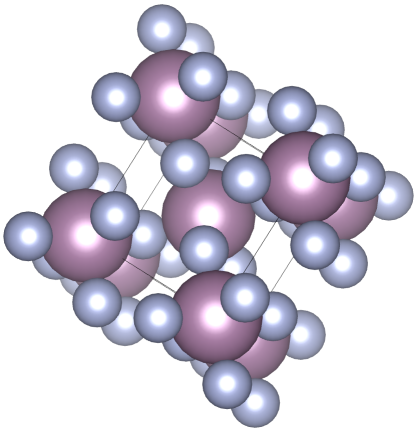

(b) MoN_2

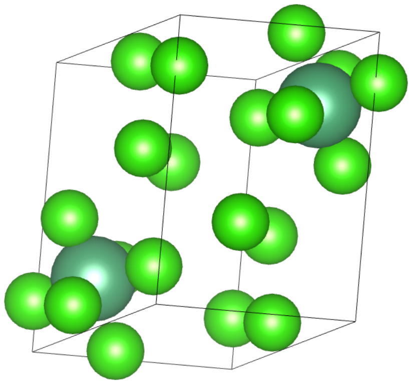

(c) $NbCl_5$

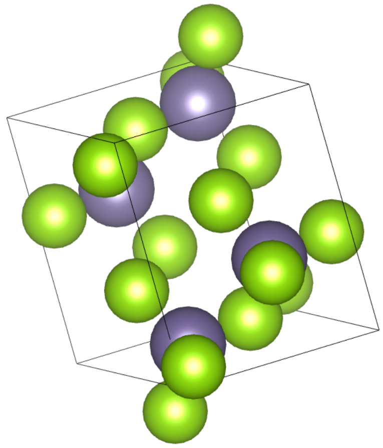

(d) $GeSe_2$

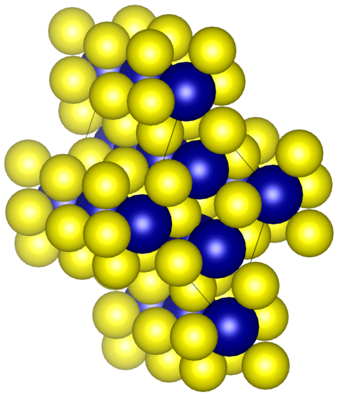

(e) Cr_2S_3

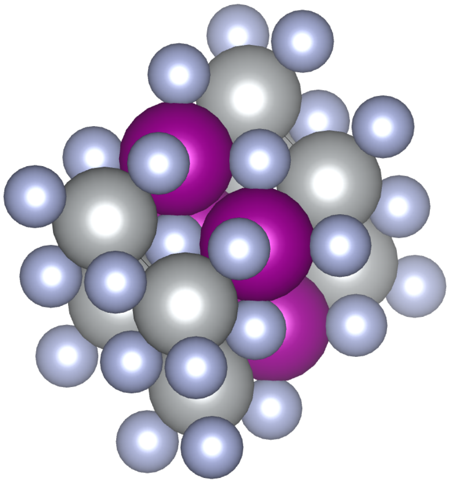

(f) $MnNiN_2$

Figure S1. Discovered binary materials by BLMM
Structures are predicted using the generated compositions and the TCSP template based crystal structure prediction algorithm.

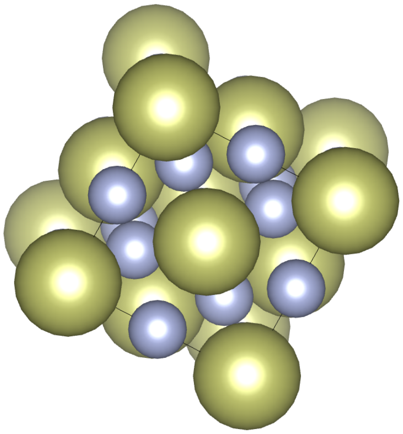

(a) IrN_3

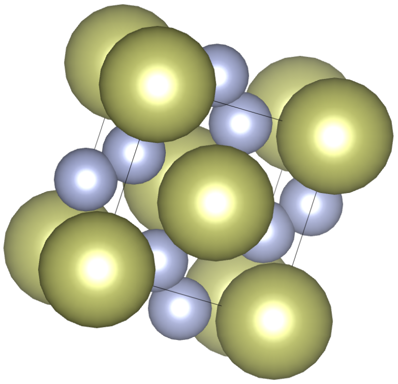

(b) IrN_2

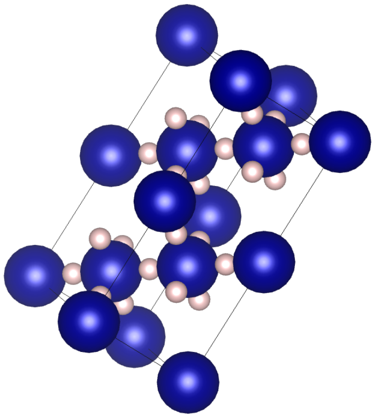

(c) CrH_3

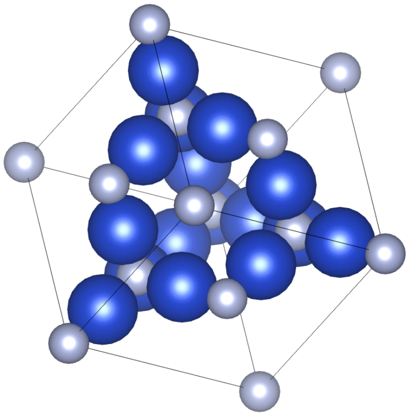

(d) Cu_2N

Figure S2. Discovered binary materials by BLMM
Structures are predicted using the generated compositions and the TCSP template based crystal structure prediction algorithm.

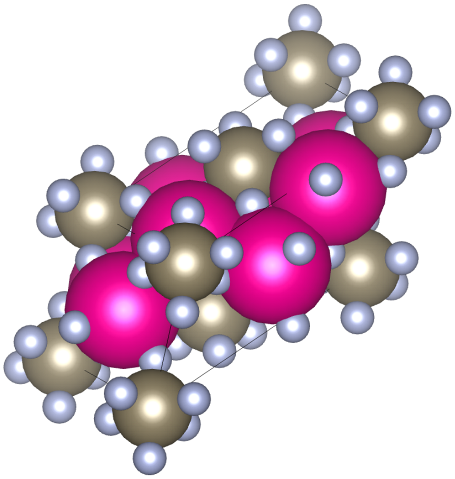

(a) Rb_3TlF_6

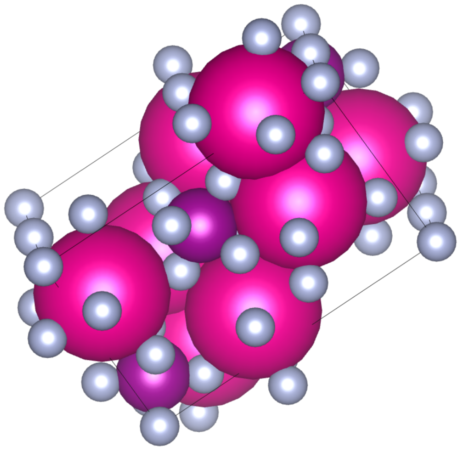

(b) Rb_2MnF_5

Figure S3. Discovered ternary materials by BLMM
Structures are predicted using the generated compositions and the TCSP template based crystal structure prediction algorithm.

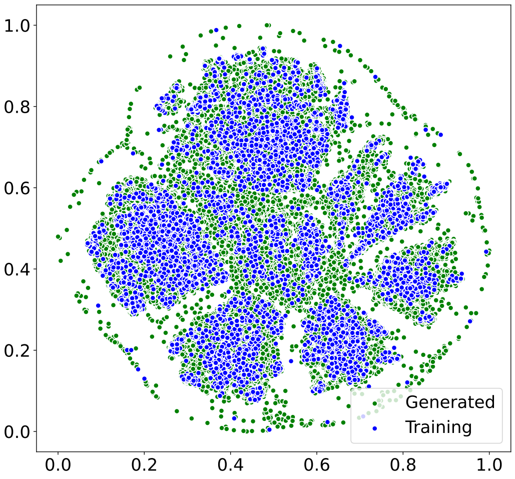

(a) Nitride distribution

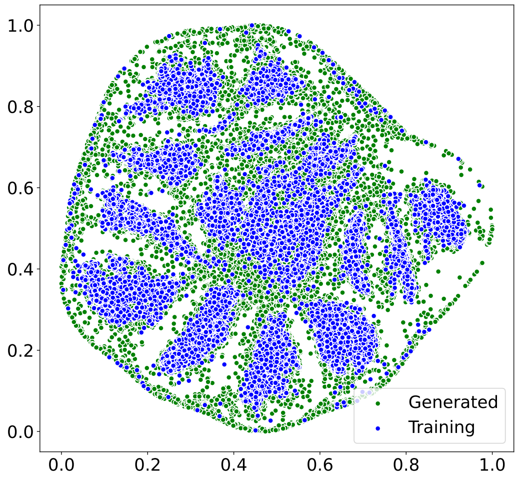

(b) Oxide distribution

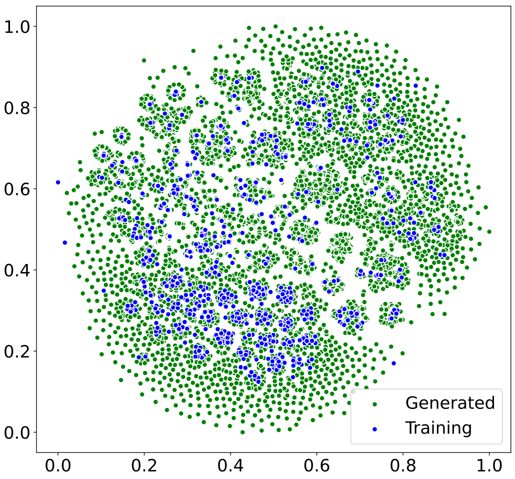

(c) ABC_3 compounds

Figure S4. Distribution of BLMM-generated nitrides, oxides, and ABC_3 compounds compared to the training ones.

Cif files of newly predicted materials

Ru4N8

```
#======================================================================
#
#----------------------------------------------------------------------
data_VESTA_phase_1

_chemical_name_common                  'Ru4 N8                                  '
_cell_length_a                         5.887278
_cell_length_b                         5.887278
_cell_length_c                         8.503650
_cell_angle_alpha                      90.000000
_cell_angle_beta                       90.000000
_cell_angle_gamma                      90.000000
_cell_volume                           294.736866
_space_group_name_H-M_alt              'P 1'
_space_group_IT_number                 1

loop_
_space_group_symop_operation_xyz
   'x, y, z'

loop_
   _atom_site_label
   _atom_site_occupancy
   _atom_site_fract_x
   _atom_site_fract_y
   _atom_site_fract_z
   _atom_site_adp_type
   _atom_site_B_iso_or_equiv
   _atom_site_type_symbol
   Ru1        1.0     -0.000000     0.000000     0.500000    Biso  1.000000 Ru
   Ru2        1.0     -0.000000     0.500000     0.750000    Biso  1.000000 Ru
   Ru3        1.0     0.500000     0.500000     0.000000    Biso  1.000000 Ru
   Ru4        1.0     0.500000     0.000000     0.250000    Biso  1.000000 Ru
   N1         1.0     0.032950     0.750000     0.625000    Biso  1.000000 N
   N2         1.0     0.750000     0.967050     0.375000    Biso  1.000000 N
   N3         1.0     0.967050     0.250000     0.625000    Biso  1.000000 N
   N4         1.0     0.250000     0.032950     0.375000    Biso  1.000000 N
   N5         1.0     0.750000     0.532950     0.875000    Biso  1.000000 N
   N6         1.0     0.250000     0.467050     0.875000    Biso  1.000000 N
   N7         1.0     0.532950     0.250000     0.125000    Biso  1.000000 N
   N8         1.0     0.467050     0.750000     0.125000    Biso  1.000000 N
```

Rb6Tl2F12

```
#======================================================================
# CRYSTAL DATA
#----------------------------------------------------------------------
data_VESTA_phase_1

_chemical_name_common                  'Rb6 Tl2 F12                             '
_cell_length_a                         6.550525
_cell_length_b                         6.732341
_cell_length_c                         11.383339
```

```
_cell_angle_alpha                      90.000000
_cell_angle_beta                       124.812607
_cell_angle_gamma                      90.000000
_cell_volume                           412.161572
_space_group_name_H-M_alt              'P 1'
_space_group_IT_number                 1

loop_
_space_group_symop_operation_xyz
   'x, y, z'

loop_
   _atom_site_label
   _atom_site_occupancy
   _atom_site_fract_x
   _atom_site_fract_y
   _atom_site_fract_z
   _atom_site_adp_type
   _atom_site_U_iso_or_equiv
   _atom_site_type_symbol
   Rb1        1.0     0.233435     0.058019     0.749672    Uiso  ? Rb
   Rb2        1.0     0.766565     0.941981     0.250328    Uiso  ? Rb
   Rb3        1.0     0.766565     0.558019     0.750328    Uiso  ? Rb
   Rb4        1.0     0.233435     0.441981     0.249672    Uiso  ? Rb
   Rb5        1.0     0.500000     0.000000     0.500000    Uiso  ? Rb
   Rb6        1.0     0.500000     0.500000     0.000000    Uiso  ? Rb
   Tl1        1.0     0.000000     0.000000     0.000000    Uiso  ? Tl
   Tl2        1.0     0.000000     0.500000     0.500000    Uiso  ? Tl
   F1         1.0     0.103646     0.276087     0.936418    Uiso  ? F
   F2         1.0     0.896354     0.723913     0.063582    Uiso  ? F
   F3         1.0     0.896354     0.776087     0.563582    Uiso  ? F
   F4         1.0     0.103646     0.223913     0.436418    Uiso  ? F
   F5         1.0     0.220222     0.678586     0.444128    Uiso  ? F
   F6         1.0     0.779778     0.321414     0.555872    Uiso  ? F
   F7         1.0     0.779778     0.178586     0.055873    Uiso  ? F
   F8         1.0     0.220222     0.821414     0.944128    Uiso  ? F
   F9         1.0     0.335214     0.043777     0.223022    Uiso  ? F
   F10        1.0     0.664786     0.956223     0.776978    Uiso  ? F
   F11        1.0     0.664786     0.543777     0.276978    Uiso  ? F
   F12        1.0     0.335214     0.456223     0.723022    Uiso  ? F
```

Rb8Mn4F20

```
#======================================================================
#
#----------------------------------------------------------------------
data_VESTA_phase_1

_chemical_name_common                  'Rb8 Mn4 F20                          '
_cell_length_a                         7.686541
_cell_length_b                         5.924994
_cell_length_c                         12.154552
_cell_angle_alpha                      90.000000
_cell_angle_beta                       90.000000
_cell_angle_gamma                      90.000000
_cell_volume                           553.551254
_space_group_name_H-M_alt              'P 1'
```

```
_space_group_IT_number                 1

loop_
_space_group_symop_operation_xyz
   'x, y, z'

loop_
   _atom_site_label
   _atom_site_occupancy
   _atom_site_fract_x
   _atom_site_fract_y
   _atom_site_fract_z
   _atom_site_adp_type
   _atom_site_B_iso_or_equiv
   _atom_site_type_symbol
   Rb1        1.0     0.018526     0.250000     0.222730    Biso  1.000000 Rb
   Rb2        1.0     0.981474     0.750000     0.777270    Biso  1.000000 Rb
   Rb3        1.0     0.481474     0.750000     0.722730    Biso  1.000000 Rb
   Rb4        1.0     0.518526     0.250000     0.277270    Biso  1.000000 Rb
   Rb5        1.0     0.149009     0.750000     0.418222    Biso  1.000000 Rb
   Rb6        1.0     0.850991     0.250000     0.581778    Biso  1.000000 Rb
   Rb7        1.0     0.350991     0.250000     0.918222    Biso  1.000000 Rb
   Rb8        1.0     0.649009     0.750000     0.081778    Biso  1.000000 Rb
   Mn1        1.0     0.158804     0.750000     0.060435    Biso  1.000000 Mn
   Mn2        1.0     0.841196     0.250000     0.939565    Biso  1.000000 Mn
   Mn3        1.0     0.341196     0.250000     0.560435    Biso  1.000000 Mn
   Mn4        1.0     0.658804     0.750000     0.439565    Biso  1.000000 Mn
   F1         1.0     0.190487     0.012618     0.616060    Biso  1.000000 F
   F2         1.0     0.809513     0.987382     0.383940    Biso  1.000000 F
   F3         1.0     0.309513     0.987382     0.116060    Biso  1.000000 F
   F4         1.0     0.690487     0.012618     0.883940    Biso  1.000000 F
   F5         1.0     0.690487     0.487382     0.883940    Biso  1.000000 F
   F6         1.0     0.309513     0.512618     0.116060    Biso  1.000000 F
   F7         1.0     0.809513     0.512618     0.383940    Biso  1.000000 F
   F8         1.0     0.190487     0.487382     0.616060    Biso  1.000000 F
   F9         1.0     -0.000000     -0.000000     0.000000    Biso  1.000000 F
   F10        1.0     0.500000     -0.000000     0.500000    Biso  1.000000 F
   F11        1.0     0.500000     0.500000     0.500000    Biso  1.000000 F
   F12        1.0     -0.000000     0.500000     0.000000    Biso  1.000000 F
   F13        1.0     0.020697     0.750000     0.187452    Biso  1.000000 F
   F14        1.0     0.979303     0.250000     0.812548    Biso  1.000000 F
   F15        1.0     0.479303     0.250000     0.687452    Biso  1.000000 F
   F16        1.0     0.520697     0.750000     0.312548    Biso  1.000000 F
   F17        1.0     0.223662     0.250000     0.421802    Biso  1.000000 F
   F18        1.0     0.776338     0.750000     0.578198    Biso  1.000000 F
   F19        1.0     0.276338     0.750000     0.921802    Biso  1.000000 F
   F20        1.0     0.723662     0.250000     0.078198    Biso  1.000000 F
```

Nb2Cl10

```
#======================================================================
#
#----------------------------------------------------------------------
data_VESTA_phase_1

_chemical_name_common                  'Nb2 Cl10                              '
_cell_length_a                         6.898922
_cell_length_b                         7.441658
```

```
_cell_length_c                          9.970851
_cell_angle_alpha                       109.964958
_cell_angle_beta                        90.882240
_cell_angle_gamma                       116.176338
_cell_volume                            423.342526
_space_group_name_H-M_alt               'P 1'
_space_group_IT_number                  1

loop_
_space_group_symop_operation_xyz
   'x, y, z'

loop_
   _atom_site_label
   _atom_site_occupancy
   _atom_site_fract_x
   _atom_site_fract_y
   _atom_site_fract_z
   _atom_site_adp_type
   _atom_site_B_iso_or_equiv
   _atom_site_type_symbol
   Nb1        1.0     0.099240     0.707109     0.210828    Biso  1.000000 Nb
   Nb2        1.0     0.900760     0.292891     0.789172    Biso  1.000000 Nb
   Cl1        1.0     0.064996     0.327027     0.596405    Biso  1.000000 Cl
   Cl2        1.0     0.935004     0.672973     0.403595    Biso  1.000000 Cl
   Cl3        1.0     0.104568     0.147989     0.855034    Biso  1.000000 Cl
   Cl4        1.0     0.895432     0.852011     0.144966    Biso  1.000000 Cl
   Cl5        1.0     0.214294     0.665117     0.961145    Biso  1.000000 Cl
   Cl6        1.0     0.785706     0.334883     0.038855    Biso  1.000000 Cl
   Cl7        1.0     0.279949     0.516185     0.232752    Biso  1.000000 Cl
   Cl8        1.0     0.720051     0.483815     0.767248    Biso  1.000000 Cl
   Cl9        1.0     0.408237     0.036362     0.314641    Biso  1.000000 Cl
   Cl10       1.0     0.591763     0.963638     0.685359    Biso  1.000000 Cl
```

Mo2N4

```
#======================================================================
#
#----------------------------------------------------------------------
data_VESTA_phase_1

_chemical_name_common                   'Mo2 N4                           '
_cell_length_a                          4.855452
_cell_length_b                          4.891908
_cell_length_c                          2.928741
_cell_angle_alpha                       90.000000
_cell_angle_beta                        90.000000
_cell_angle_gamma                       90.000000
_cell_volume                            69.564690
_space_group_name_H-M_alt               'P 1'
_space_group_IT_number                  1

loop_
_space_group_symop_operation_xyz
   'x, y, z'

loop_
   _atom_site_label
```

```
   _atom_site_occupancy
   _atom_site_fract_x
   _atom_site_fract_y
   _atom_site_fract_z
   _atom_site_adp_type
   _atom_site_B_iso_or_equiv
   _atom_site_type_symbol
   Mo1        1.0     -0.000000     0.000000     0.000000    Biso  1.000000 Mo
   Mo2        1.0     0.500000     0.500000     0.500000    Biso  1.000000 Mo
   N1         1.0     0.296747     0.307345     -0.000000    Biso  1.000000 N
   N2         1.0     0.703253     0.692655     0.000000    Biso  1.000000 N
   N3         1.0     0.796747     0.192655     0.500000    Biso  1.000000 N
   N4         1.0     0.203253     0.807345     0.500000    Biso  1.000000 N
```

MnNiN2

```
#======================================================================
#
#----------------------------------------------------------------------
data_VESTA_phase_1

_chemical_name_common                  'Mn1 Ni1 N2                            '
_cell_length_a                         2.779513
_cell_length_b                         2.779513
_cell_length_c                         4.886258
_cell_angle_alpha                      90.000000
_cell_angle_beta                       90.000000
_cell_angle_gamma                      120.000000
_cell_volume                           32.692232
_space_group_name_H-M_alt              'P 1'
_space_group_IT_number                 1

loop_
_space_group_symop_operation_xyz
   'x, y, z'

loop_
   _atom_site_label
   _atom_site_occupancy
   _atom_site_fract_x
   _atom_site_fract_y
   _atom_site_fract_z
   _atom_site_adp_type
   _atom_site_B_iso_or_equiv
   _atom_site_type_symbol
   Mn1        1.0     -0.000000     0.000000     0.500000    Biso  1.000000 Mn
   Ni1        1.0     -0.000000     0.000000     0.000000    Biso  1.000000 Ni
   N1         1.0     0.333333     0.666667     0.739100    Biso  1.000000 N
   N2         1.0     0.666667     0.333333     0.260900    Biso  1.000000 N
```

Ir4N12

```
#======================================================================
#
#----------------------------------------------------------------------
data_VESTA_phase_1
```

```
_chemical_name_common                  'Ir4 N12                               '
_cell_length_a                         5.153458
_cell_length_b                         5.153458
_cell_length_c                         5.153458
_cell_angle_alpha                      90.000000
_cell_angle_beta                       90.000000
_cell_angle_gamma                      90.000000
_cell_volume                           136.866214
_space_group_name_H-M_alt              'P 1'
_space_group_IT_number                 1

loop_
_space_group_symop_operation_xyz
   'x, y, z'

loop_
   _atom_site_label
   _atom_site_occupancy
   _atom_site_fract_x
   _atom_site_fract_y
   _atom_site_fract_z
   _atom_site_adp_type
   _atom_site_B_iso_or_equiv
   _atom_site_type_symbol
   Ir1        1.0     0.000000     0.000000     -0.000000    Biso  1.000000 Ir
   Ir2        1.0     0.500000     0.500000     -0.000000    Biso  1.000000 Ir
   Ir3        1.0     0.500000     0.000000     0.500000    Biso  1.000000 Ir
   Ir4        1.0     -0.000000     0.500000     0.500000    Biso  1.000000 Ir
   N1         1.0     0.250000     0.250000     0.250000    Biso  1.000000 N
   N2         1.0     0.750000     0.750000     0.750000    Biso  1.000000 N
   N3         1.0     0.750000     0.250000     0.250000    Biso  1.000000 N
   N4         1.0     0.250000     0.750000     0.750000    Biso  1.000000 N
   N5         1.0     0.750000     0.750000     0.250000    Biso  1.000000 N
   N6         1.0     0.250000     0.250000     0.750000    Biso  1.000000 N
   N7         1.0     0.250000     0.750000     0.250000    Biso  1.000000 N
   N8         1.0     0.750000     0.250000     0.750000    Biso  1.000000 N
   N9         1.0     -0.000000     0.000000     0.500000    Biso  1.000000 N
   N10        1.0     0.500000     0.000000     -0.000000    Biso  1.000000 N
   N11        1.0     -0.000000     0.500000     -0.000000    Biso  1.000000 N
   N12        1.0     0.500000     0.500000     0.500000    Biso  1.000000 N
```

Ir2N4

```
#======================================================================
#
#----------------------------------------------------------------------
data_VESTA_phase_1

_chemical_name_common                  'Ir2 N4                                '
_cell_length_a                         4.538286
_cell_length_b                         4.538286
_cell_length_c                         2.737344
_cell_angle_alpha                      90.000000
_cell_angle_beta                       90.000000
_cell_angle_gamma                      90.000000
_cell_volume                           56.378452
_space_group_name_H-M_alt              'P 1'
```

```
_space_group_IT_number                 1

loop_
_space_group_symop_operation_xyz
   'x, y, z'

loop_
   _atom_site_label
   _atom_site_occupancy
   _atom_site_fract_x
   _atom_site_fract_y
   _atom_site_fract_z
   _atom_site_adp_type
   _atom_site_B_iso_or_equiv
   _atom_site_type_symbol
   Ir1        1.0     -0.000000     -0.000000     -0.000000    Biso  1.000000 Ir
   Ir2        1.0     0.500000     0.500000     -0.000000    Biso  1.000000 Ir
   N1         1.0     0.100881     0.399119     0.500000    Biso  1.000000 N
   N2         1.0     0.899119     0.600881     0.500000    Biso  1.000000 N
   N3         1.0     0.600881     0.100881     0.500000    Biso  1.000000 N
   N4         1.0     0.399119     0.899119     0.500000    Biso  1.000000 N
```

Cr6H18

```
#======================================================================
#
#----------------------------------------------------------------------
data_VESTA_phase_1

_chemical_name_common                  'Cr6 H18                              '
_cell_length_a                         4.833354
_cell_length_b                         4.833354
_cell_length_c                         11.817400
_cell_angle_alpha                      90.000000
_cell_angle_beta                       90.000000
_cell_angle_gamma                      120.000000
_cell_volume                           239.083570
_space_group_name_H-M_alt              'P 1'
_space_group_IT_number                 1

loop_
_space_group_symop_operation_xyz
   'x, y, z'

loop_
   _atom_site_label
   _atom_site_occupancy
   _atom_site_fract_x
   _atom_site_fract_y
   _atom_site_fract_z
   _atom_site_adp_type
   _atom_site_B_iso_or_equiv
   _atom_site_type_symbol
   Cr1        1.0     0.000000     -0.000000     0.000000    Biso  1.000000 Cr
   Cr2        1.0     0.333333     0.666667     0.166667    Biso  1.000000 Cr
   Cr3        1.0     0.666667     0.333333     0.333333    Biso  1.000000 Cr
   Cr4        1.0     0.000000     -0.000000     0.500000    Biso  1.000000 Cr
```

```
   Cr5        1.0     0.333333     0.666667     0.666667    Biso  1.000000 Cr
   Cr6        1.0     0.666667     0.333333     0.833333    Biso  1.000000 Cr
   H1         1.0     0.000000     0.501247     0.250000    Biso  1.000000 H
   H2         1.0    -0.000000     0.498753     0.750000    Biso  1.000000 H
   H3         1.0     0.498753     0.498753     0.250000    Biso  1.000000 H
   H4         1.0     0.501247     0.501247     0.750000    Biso  1.000000 H
   H5         1.0     0.501247     -0.000000     0.250000    Biso  1.000000 H
   H6         1.0     0.498753     -0.000000     0.750000    Biso  1.000000 H
   H7         1.0     0.834580     0.666667     0.916667    Biso  1.000000 H
   H8         1.0     0.832086     0.666667     0.416667    Biso  1.000000 H
   H9         1.0     0.832086     0.165420     0.916667    Biso  1.000000 H
   H10        1.0     0.834580     0.167914     0.416667    Biso  1.000000 H
   H11        1.0     0.333333     0.167914     0.916667    Biso  1.000000 H
   H12        1.0     0.333333     0.165420     0.416667    Biso  1.000000 H
   H13        1.0     0.666667     0.834580     0.583333    Biso  1.000000 H
   H14        1.0     0.666667     0.832086     0.083333    Biso  1.000000 H
   H15        1.0     0.165420     0.832086     0.583333    Biso  1.000000 H
   H16        1.0     0.167914     0.834580     0.083333    Biso  1.000000 H
   H17        1.0     0.167914     0.333333     0.583333    Biso  1.000000 H
   H18        1.0     0.165420     0.333333     0.083333    Biso  1.000000 H
```

Cu16N8

```
#======================================================================
#
#----------------------------------------------------------------------
data_VESTA_phase_1

_chemical_name_common                  'Cu16 N8                              '
_cell_length_a                         8.401162
_cell_length_b                         8.401162
_cell_length_c                         8.401162
_cell_angle_alpha                      90.000000
_cell_angle_beta                       90.000000
_cell_angle_gamma                      90.000000
_cell_volume                           592.950037
_space_group_name_H-M_alt              'P 1'
_space_group_IT_number                 1

loop_
_space_group_symop_operation_xyz
   'x, y, z'

loop_
   _atom_site_label
   _atom_site_occupancy
   _atom_site_fract_x
   _atom_site_fract_y
   _atom_site_fract_z
   _atom_site_adp_type
   _atom_site_B_iso_or_equiv
   _atom_site_type_symbol
   Cu1        1.0     0.125000     0.125000     0.125000    Biso  1.000000 Cu
   Cu2        1.0     0.125000     0.875000     0.875000    Biso  1.000000 Cu
   Cu3        1.0     0.875000     0.875000     0.125000    Biso  1.000000 Cu
   Cu4        1.0     0.375000     0.625000     0.875000    Biso  1.000000 Cu
   Cu5        1.0     0.125000     0.625000     0.625000    Biso  1.000000 Cu
   Cu6        1.0     0.875000     0.125000     0.875000    Biso  1.000000 Cu
```

```
   Cu7        1.0     0.375000     0.875000     0.625000    Biso  1.000000 Cu
   Cu8        1.0     0.625000     0.625000     0.125000    Biso  1.000000 Cu
   Cu9        1.0     0.625000     0.375000     0.875000    Biso  1.000000 Cu
   Cu10       1.0     0.375000     0.375000     0.125000    Biso  1.000000 Cu
   Cu11       1.0     0.625000     0.125000     0.625000    Biso  1.000000 Cu
   Cu12       1.0     0.875000     0.375000     0.625000    Biso  1.000000 Cu
   Cu13       1.0     0.625000     0.875000     0.375000    Biso  1.000000 Cu
   Cu14       1.0     0.875000     0.625000     0.375000    Biso  1.000000 Cu
   Cu15       1.0     0.375000     0.125000     0.375000    Biso  1.000000 Cu
   Cu16       1.0     0.125000     0.375000     0.375000    Biso  1.000000 Cu
   N1         1.0     -0.000000     0.000000     -0.000000    Biso  1.000000 N
   N2         1.0     0.250000     0.750000     0.750000    Biso  1.000000 N
   N3         1.0     0.500000     0.500000     -0.000000    Biso  1.000000 N
   N4         1.0     0.750000     0.250000     0.750000    Biso  1.000000 N
   N5         1.0     0.500000     0.000000     0.500000    Biso  1.000000 N
   N6         1.0     0.750000     0.750000     0.250000    Biso  1.000000 N
   N7         1.0     -0.000000     0.500000     0.500000    Biso  1.000000 N
   N8         1.0     0.250000     0.250000     0.250000    Biso  1.000000 N
```

Ge4Se8

```
#======================================================================
#
#----------------------------------------------------------------------
data_VESTA_phase_1

_chemical_name_common                  'Ge4 Se8                              '
_cell_length_a                         7.572346
_cell_length_b                         8.443511
_cell_length_c                         6.507539
_cell_angle_alpha                      90.000000
_cell_angle_beta                       109.820717
_cell_angle_gamma                      90.000000
_cell_volume                           391.424765
_space_group_name_H-M_alt              'P 1'
_space_group_IT_number                 1

loop_
_space_group_symop_operation_xyz
   'x, y, z'

loop_
   _atom_site_label
   _atom_site_occupancy
   _atom_site_fract_x
   _atom_site_fract_y
   _atom_site_fract_z
   _atom_site_adp_type
   _atom_site_B_iso_or_equiv
   _atom_site_type_symbol
   Ge1        1.0     0.126823     0.135156     0.137690    Biso  1.000000 Ge
   Ge2        1.0     0.873177     0.864844     0.862310    Biso  1.000000 Ge
   Ge3        1.0     0.873177     0.635156     0.362310    Biso  1.000000 Ge
   Ge4        1.0     0.126823     0.364844     0.637690    Biso  1.000000 Ge
   Se1        1.0     0.209647     0.618497     0.526350    Biso  1.000000 Se
   Se2        1.0     0.790353     0.381503     0.473650    Biso  1.000000 Se
   Se3        1.0     0.790353     0.118497     0.973650    Biso  1.000000 Se
   Se4        1.0     0.209647     0.881503     0.026350    Biso  1.000000 Se
```

```
   Se5        1.0     0.258882     0.133922     0.530310    Biso  1.000000 Se
   Se6        1.0     0.741118     0.866078     0.469690    Biso  1.000000 Se
   Se7        1.0     0.741118     0.633922     0.969690    Biso  1.000000 Se
   Se8        1.0     0.258882     0.366078     0.030310    Biso  1.000000 Se
```

Cr8S12

```
#======================================================================
#
#----------------------------------------------------------------------
data_VESTA_phase_1

_chemical_name_common                  'Cr8 S12                                  '
_cell_length_a                         6.023805
_cell_length_b                         6.023805
_cell_length_c                         11.255604
_cell_angle_alpha                      90.000000
_cell_angle_beta                       90.000000
_cell_angle_gamma                      120.000000
_cell_volume                           353.704992
_space_group_name_H-M_alt              'P 1'
_space_group_IT_number                 1

loop_
_space_group_symop_operation_xyz
   'x, y, z'

loop_
   _atom_site_label
   _atom_site_occupancy
   _atom_site_fract_x
   _atom_site_fract_y
   _atom_site_fract_z
   _atom_site_adp_type
   _atom_site_B_iso_or_equiv
   _atom_site_type_symbol
   Cr1        1.0     0.333333     0.666667     0.517277    Biso  1.000000 Cr
   Cr2        1.0     0.666667     0.333333     0.482723    Biso  1.000000 Cr
   Cr3        1.0     0.333333     0.666667     0.982723    Biso  1.000000 Cr
   Cr4        1.0     0.666667     0.333333     0.017277    Biso  1.000000 Cr
   Cr5        1.0     0.000000     0.000000     0.000000    Biso  1.000000 Cr
   Cr6        1.0     0.000000     0.000000     0.500000    Biso  1.000000 Cr
   Cr7        1.0     0.333333     0.666667     0.250000    Biso  1.000000 Cr
   Cr8        1.0     0.666667     0.333333     0.750000    Biso  1.000000 Cr
   S1         1.0     0.021485     0.346052     0.383418    Biso  1.000000 S
   S2         1.0     0.978515     0.653948     0.616582    Biso  1.000000 S
   S3         1.0     0.653948     0.675433     0.383418    Biso  1.000000 S
   S4         1.0     0.346052     0.324567     0.616582    Biso  1.000000 S
   S5         1.0     0.324567     0.978515     0.383418    Biso  1.000000 S
   S6         1.0     0.675433     0.021485     0.616582    Biso  1.000000 S
   S7         1.0     0.653948     0.978515     0.116582    Biso  1.000000 S
   S8         1.0     0.346052     0.021485     0.883418    Biso  1.000000 S
   S9         1.0     0.324567     0.346052     0.116582    Biso  1.000000 S
   S10        1.0     0.675433     0.653948     0.883418    Biso  1.000000 S
   S11        1.0     0.021485     0.675433     0.116582    Biso  1.000000 S
   S12        1.0     0.978515     0.324567     0.883418    Biso  1.000000 S
```
